# Electroacupuncture pre-treatment alleviates sepsis-induced cardiac inflammation and dysfunction by inhibiting the calpain-2/STAT3 pathway

**DOI:** 10.3389/fphys.2022.961909

**Published:** 2022-09-07

**Authors:** Xuqing Li, Li Wang, Xinwang Ying, Yujun Zheng, Qianqian Tan, Xiaolan Yu, Jiahong Gong, Ming Li, Xiaofeng Deng, Guanhu Yang, Shengcun Li, Songhe Jiang

**Affiliations:** ^1^ Rehabilitation Medicine Center, The Second Affiliated Hospital and Yuying Children’s Hospital of Wenzhou Medical University, Wenzhou, Zhejiang, China; ^2^ Integrative and Optimized Medicine Research Center, China-USA Institute for Acupuncture and Rehabilitation, Wenzhou Medical University, Wenzhou, Zhejiang, China; ^3^ Wenzhou Sports School, Wenzhou Sports Science Research Institute, Wenzhou, Zhejiang, China; ^4^ Oujiang Laboratory (Zhejiang Lab for Regenerative Medicine, Vision and Brain Health), School of Pharmaceutical Science, Wenzhou Medical University, Wenzhou, Zhejiang, China

**Keywords:** electroacupuncture, myocardial inflammation, calpain-2, stat3, heart

## Abstract

Electroacupuncture (EA) has both anti-inflammatory and cardio-protective effects. Activation of calpain pathway is involved in several myocardiopathy. In sepsis, the role of calpain-2-regulated STAT3 in cardio-protective mechanism of electroacupuncture remains unclear. In this study, we aimed to elucidate the mechanism by which electroacupuncture reduces cardiac inflammation and apoptosis and improves cardiac function during sepsis. Electroacupuncture pretreatment for 7 days was applied in septic cardiomyopathy model induced by lipopolysaccharide (LPS). lipopolysaccharide-induced sepsis was associated with a dramatically systemic inflammation and cardiac dysfunction, which was alleviated by electroacupuncture pre-treatment. Lipopolysaccharide resulted in increases of pro-inflammatory factors (TNF-α,IL1βand IL-6) and apoptosis (TUNEL staining and BAX/Bcl2) via activation of calpain-2/STAT3 pathway.Electroacupuncture pre-treatment inhibited LPS-induced activation of cardiac calpain-2/STAT3 signalling and ameliorated inflammatory and apoptosis. Additionally, inhibition of calpain-2 expression using the corresponding siRNA decreased the Phosphorylation of STAT3,pro-inflammatory factors and apoptosis in lipopolysaccharide- treated cardiomyocytes, confirming that calpain-2 activated p-STAT3 participate in septic cardiomyopathy. Furthermore, suppression of STAT3 by stattic enhanced anti-inflammatory and anti-apoptosis effects of electroacupuncture. These findings reveal mechanisms of electroacupuncture preconditioning protection against cardiac inflammation and apoptosis in sepsis mouse via calpain-2/STAT3 pathway and may provide novel targets for clinical treatments of the sepsis-induced cardiac dysfunction.

## Introduction

Sepsis is a common cause of mortality worldwide, and its incidence is steadily increasing (Tindal et al., 2021). Acute systemic and excessive inflammation response is a critical feature in patients with sepsis (Karampela et al., 2019). Subsequently, local cardiac inflammation will promote oxidative stress, autophagy disorder, and apoptotic damage, which may result in irreversible myocardial morphological and functional alterations (Hollenberg and Singer, 2021). As septic common complication, inflammation-induced myocardial injury is closely associated with mortality and prognosis in patients (Tsolaki et al., 2017). Among various treatments for septic cardiomyopathy, blocking the inflammatory cytokine storm may be an effective strategy because it can achieve dual protection via both systemic and cardiac pathways (Zechendorf et al., 2020).

Acupuncture is an important therapeutic approach in traditional Chinese medicine. Electroacupuncture (EA) provide strong and continuous stimuli to improve the efficacy of treatment compared with conventional acupuncture. Therefore, EA is widely used in clinical settings. Importantly, EA strengthens the cardiac function and exerts both anti-apoptosis (Xiao et al., 2020)anti-inflammatory effects (Wang et al., 2021). Indeed, acupuncture can reduce the production of pro-inflammatory mediators and improved the survival in LPS-induced septic mice (Liu S et al., 2021). However, the mechanism of EA on septic cardiomyopathy is still poorly understood. Generally, acupuncture at PC6(Neiguan) strengthens the cardiac function (Tsou et al., 2004; Wang et al., 2021; Hong et al., 2022) and acupuncture at ST36 (Zusanli) counteracts inflammation (Chen et al., 2019; Lv et al., 2022). Therefore, we adopted this clinical combination as an intervention means and conducted a mechanism research in this study.

Lipopolysaccharide (LPS)-induced endotoxaemia is widely used for sepsis research. In a previous study, LPS treatment resulted in a 4.1-fold increase calpain activity in myocardial tissue (Luo et al., 2019). Furthermore, LPS-mediated upregulation of TNF-α expression was abrogated after transfection with calpain-1 siRNA or various pharmacological calpain inhibitors (Li et al., 2009). Calpain-1 and calpain-2 are the main members of the calpain family, and calpain-2 is more abundant in the heart than calpain-2. However, the role of calpain-2 in sepsis-associated cardiomyopathy is largely unknown. In addition, signal transducer and activator of transcription 3 (STAT3) as a molecule that promotes the survival and inflammation in heart, is closely related to calpain in cardiomyocyte biological function. Thus, in this study, we aimed to investigate the protective effect of EA pre-treatment against sepsis cardiomyopathy and the potential role of calpain-2/STAT3 signalling in process ofinflammation and apoptosis. The findings of this study may provide new pharmacological molecular targets for clinical sepsis patients.

## Materials and methods

### Animal model of sepsis

All animal experiments were approved by the Institutional Animal Care and Use Committee of Wenzhou Medical University and were performed in accordance with the National Institute of Health’s Guide for the Care and Use of Laboratory Animals. Male C57BL/6 mice, aged 8–9°weeks, were purchased from Model Animal Research Centre of Wenzhou Medical University. The mice were housed at a constant temperature of 22°C in a 12-/12-h light/dark cycle; they had free access to regular rodent chow and tap water.

A septic cardiomyopathy model was established via an intraperitoneal injection of 6 mg/kg lipopolysaccharide (LPS, Sigma 055: B5, L2880), as described previously, and cardiac function, apoptosis, and serum inflammatory markers were assessed after 8 h of LPS treatment (Honda et al., 2019).

### Electroacupuncture pre-treatment and inhibitor injection

**FIGURE 1:**
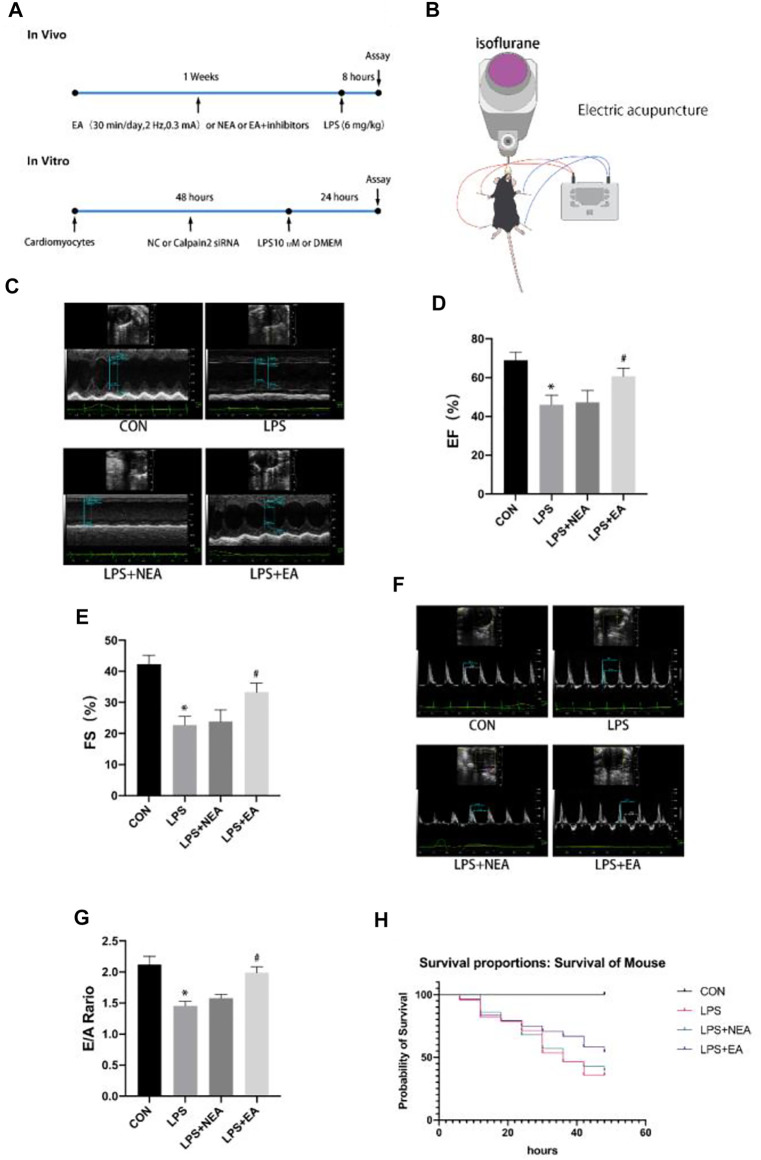
EA pre-treatment attenuates LPS-induced cardiac dysfunction. **(A)** Schematic diagram of the experimental protocols. **(B)** Schematic representation of EA treatment in mice. NEA group was the same current loop but the acupoints at digits from corresponding limbs. **(C)** Representative images of M-mode echocardiography in the LPS + EA pre-treatment group. **(D)** Ejection fraction (EF). **(E)** Fractional shortening (FS). **(F)** Representative mitral E/A image on Doppler echocardiography. **(G)** E/A ratio. N = 4 per group. EA, electroacupuncture; NEA, EA in non-acupoint; LPS, lipopolysaccharide. *p < 0.05 vs. control group, ^#^ *p* < 0.05 vs. LPS group. **(H)** Survival curve during 48 h,N = 8 in the CON group,N = 28 in the LPS group, N = 28 in the LPS + NEA group, and N = 24 in the LPS + EA group.

The mice were divided into the following four groups, with 8–16 mice per group: control, LPS, LPS with non-EA (LPS + NEA), and LPS with EA (LPS + EA). Briefly, the mice were anaesthetised and maintained by inhalation of 1.5% isoflurane using an isoflurane vaporiser. The needles connected to the electrodes were inserted 1–2 mm deep in the muscle at the Neiguan acupoint (PC6) and zusanli (ST36) of both forelimbs and hindlimbs (Figure 1B) (Wang et al., 2021). The mice were stimulated at a density of 0.3 mA and a frequency of 2 Hz for 30 min using an electric stimulation device (Hans-200e; Jisheng Medical Device, Jiangsu, China) once a day for 7 days. The mice were intraperitoneally injected with LPS within 30 min of the last EA treatment. The mice in the control group were injected with an equivalent dose of normal saline. The mice in the LPS + Non-acupoint EA (NEA) group were anaesthetised for 30 min, and the needle was inserted about 1 mm into the digits (between the first and second) at both upper and lower limbs, to avoid the effects of isoflurane, stress and currents (Wang et al., 2018). The mice in the LPS + EA + Stattic group received STAT3 inhibitor Stattic (5 mg/kg) 30 min before EA treatment. The inhibitor was injected intraperitoneally.

### Echocardiography

After treatment, the mice were anaesthetised with isoflurane (1%), and an animal ultrasound system Vevo2100 (VisualSonics, Canada) equipped with a 40-MHz paediatric transducer was used to analyse cardiac function (Li et al., 2021). The M-mode and 2-D parasternal short-axis scans at the level of the papillary muscles were used to assess the changes in the left ventricle (LV) end-systolic inner diameter, LV end-diastolic inner diameter, and fractional shortening (FS, %). To assess diastolic function, early diastolic mitral annular velocity (E) and late diastolic mitral annular velocity (A) were measured, and the E/A ratio was calculated. When collecting diastolic function, the heart rate of mice was maintained at 270-350 bpm, while when obtaining systolic function, the heart rate was 370-450 bpm.

### Serum inflammatory markers

Mouse sera were collected, and the levels of inflammatory markers were measured using the TNF-α (JL10484; Jianglaishengwu, Shanghai, China), IL-1β (JL18442; Jianglaishengwu) by ELISA kits, according to the manufacturer’s instructions.

### Quantitative polymerase chain reaction

The total RNA was isolated from the mouse heart tissue using TRIzol reagent (TaKaRa, Dalian, China), and 1,000 ng of RNA was reverse transcribed into cDNA using the first-strand synthesis system for polymerase chain reaction (PCR) (Invitrogen, Carlsbad, CA, United States) according to the manufacturer’s protocol. The cDNA was used as a template in quantitative (qPCR) to analyse the mRNA expression levels of *Il-1β*, *Tnf-α*, and *calpain-2*.

### Western blotting

Western blotting was performed using antibodies against calpain-2 (Cat. 2,539, 1:1,000; Cell Signaling Technology), IL-6 (ab233706,1:1,000; Abcam, Cambridge, United Kingdom), TNF-α (ab183218,1:1,000; Abcam, Cambridge, United Kingdom), IL-1β (ab216995,1:1,000; Abcam, Cambridge, United Kingdom), p-STAT3 (Tyr705) (Cat. 9,145, 1:1,000; Cell Signaling Technology), STAT3 (Cat. 5,345, 1:1,000; Cell Signaling Technology), and GAPDH (Cat. 2,118, 1:5,000; Cell Signaling Technology). Next, the proteins were probed with horseradish peroxidase (HRP)-conjugated secondary antibodies and visualised using a ChemiDoc Imaging System (Bio-Rad Laboratories, Hercules, CA, United States). The relative quantity of proteins is presented as the ratio of target proteins to GAPDH.

### Immunohistochemistry

We detected pro-inflammatory factors (TNF-α and IL-1β) by immunohistochemistry as described previously (Qian et al., 2021).

### Cardiomyocyte experiment and siRNA construction

Neonatal mice (aged no more than 2 days) were euthanized and got heart. The myocardial tissue was cut in D-Hank’s buffer and digested with collagenase for 10 min. Thereafter, the cells were collected and centrifuged (1000 g, 5 min). The total cells were then cultured for 2 h to separate cardiomyocytes from fibroblasts (Li et al., 2016; Chen et al., 2021). The cell experiment data were obtained from three independent cultures. Cholesterol-conjugated siRNA for calpain-2 was purchased from Shanghai Gene Pharma Co., Ltd. (China). The sequences for Calpina-2 siRNA are as follows:

5′GGA​GAA​AGG​TTC​TCT​GCT​T3′. The cardiomyocytes were incubated with siRNA (50 nM) combined transfection reagent for 48 h in standard culture medium. LPS (10 μmol/L) was subsequently added to the culture, which was incubated for another 24 h.

### Statistical analysis

All data are presented as mean ± SD. Statistical significance was determined using a one-way analysis of variance (ANOVA). Student’s *t*-test was used to compare two groups. Statistical analyses were performed using GraphPad Prism v5.0 (GraphPad Software, San Diego, CA, United States), and the statistical significance was set at *p* < 0.05.

## Results

### Electroacupuncture pre-treatment protected cardiac function against septic cardiomyopathy

Experimental overall design (Figure 1A)and electroacupuncture treatment scheme (Figure 1B). Echocardiography was performed to evaluate the effects of EA on cardiac function following septic injury. The M-mode images were obtained to measure ejection fraction (EF) and FS to evaluate the cardiac contractile function. LPS significantly reduced EF and FS compared with the control group, whereas EA pre-treatment for seven consecutive days significantly increased EF and FS compared with the LPS group (Figures 1C–E). Similarly, the E/A ratio represented heart diastolic function was reduced in the LPS group compared with that in the CON group, but it was significantly increased in the LPS + EA group compared with LPS group (Figures 1F,G). These findings demonstrate that the LPS treatment compromised cardiac function, whereas EA pre-treatment improved cardiac function. However, cardiac function in the LPS + NEA group was not significantly altered compared with that in the LPS group, implying that the current stimulation did not affect cardiac function in the septic mice. These results revealed that EA pre-treatment alleviated LPS-induced cardiac dysfunction.

### Electroacupuncture pre-treatment increased survival and attenuated myocardial inflammatory and apoptosis in septic mice

**FIGURE 2:**
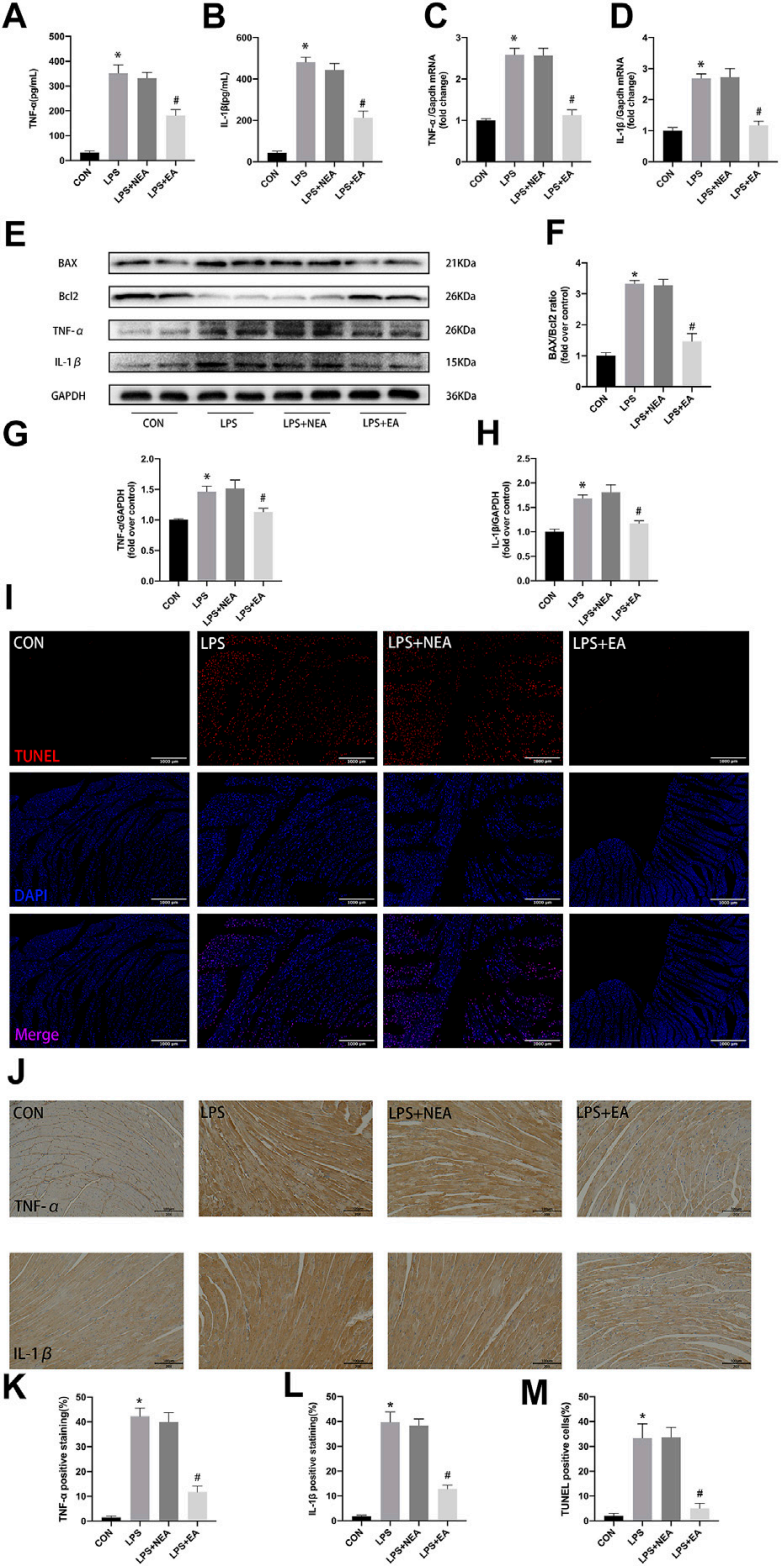
Effect of EA pre-treatment on myocardial inflammatory response and apoptosis. **(A,B)** Serum concentrations of TNF-α and IL-1β. **(C,D)** mRNA expression of *Tnf-α* and *Il-1β*. **(E)** Representative images of western blots for BAX,Bcl2,TNF-α, IL-1β, and GAPDH. **(F–H)** Quantitative results of BAX\Bcl2, TNF-α and IL-1β. **(I)** TUNEL staining, apoptosis positive cells (red), nuclei (blue).**(J)** Immunohistochemistry results of TNF-α, IL-1β. **(K,L)** Quantitative analysis for TNF-α and IL-1β. **(M)** Quantitative analysis for TUNEL postive cells. N = 4 per group. EA, electroacupuncture; NEA, EA in non-acupoint; LPS, lipopolysaccharide. *p < 0.05 vs. control group, ^#^ *p* < 0.05 vs. LPS group.

LPS triggers myocardial inflammation. An uncontrollably sustained and vigorous inflammatory response is a characteristic of severe sepsis, and it ultimately leads to death. In the present study, the LPS treatment increased the mortality of mice, whereas the EA treatment improved the survival of mice compared with control group (Figure 1H). Furthermore, sepsis led to a significant increase in plasma (Figures 2A,B)and myocardial (Figures 2C,D,G,H) levels of pro-inflammatory factors, such as TNF-α and IL-1β, compared with those in the control group, whereas the EA pre-treatment reduced inflammation compared with the LPS group (Figures 2A–F). The immunohistochemistry results showed that the levels of TNF-α and IL-1β in the heart tissue increased in response to LPS treatment compared with those in the control group, whereas the EA pre-treatment decreased the expression of these proteins in the LPS group (Figures 2J–L). These findings revealed that the myocardial inflammation caused by sepsis was inhibited by EA pre-treatment.

In addition, LPS caused the imbalance of the expression of Pro-apoptotic protein and anti-apoptotic protein in mouse heart tissue, resulting in a significant increase of BAX/Bcl2 ratio (Figures 2E,F). EA pre-treatment decreased the expression of BAX and increased the expression of Bcl2. Similarly, TUNEL staining showed that there were more positive cells in LPS treated myocardial tissue than in the CON and EA pre-treatment groups (Figures 2I–M). These results indicate that electroacupuncture pretreatment can reduce cardiomyocyte apoptosis in sepsis mice.

### Electroacupuncture regulated lipopolysaccharide-induced myocardial calpain-2/STAT3 pathway

**FIGURE 3:**
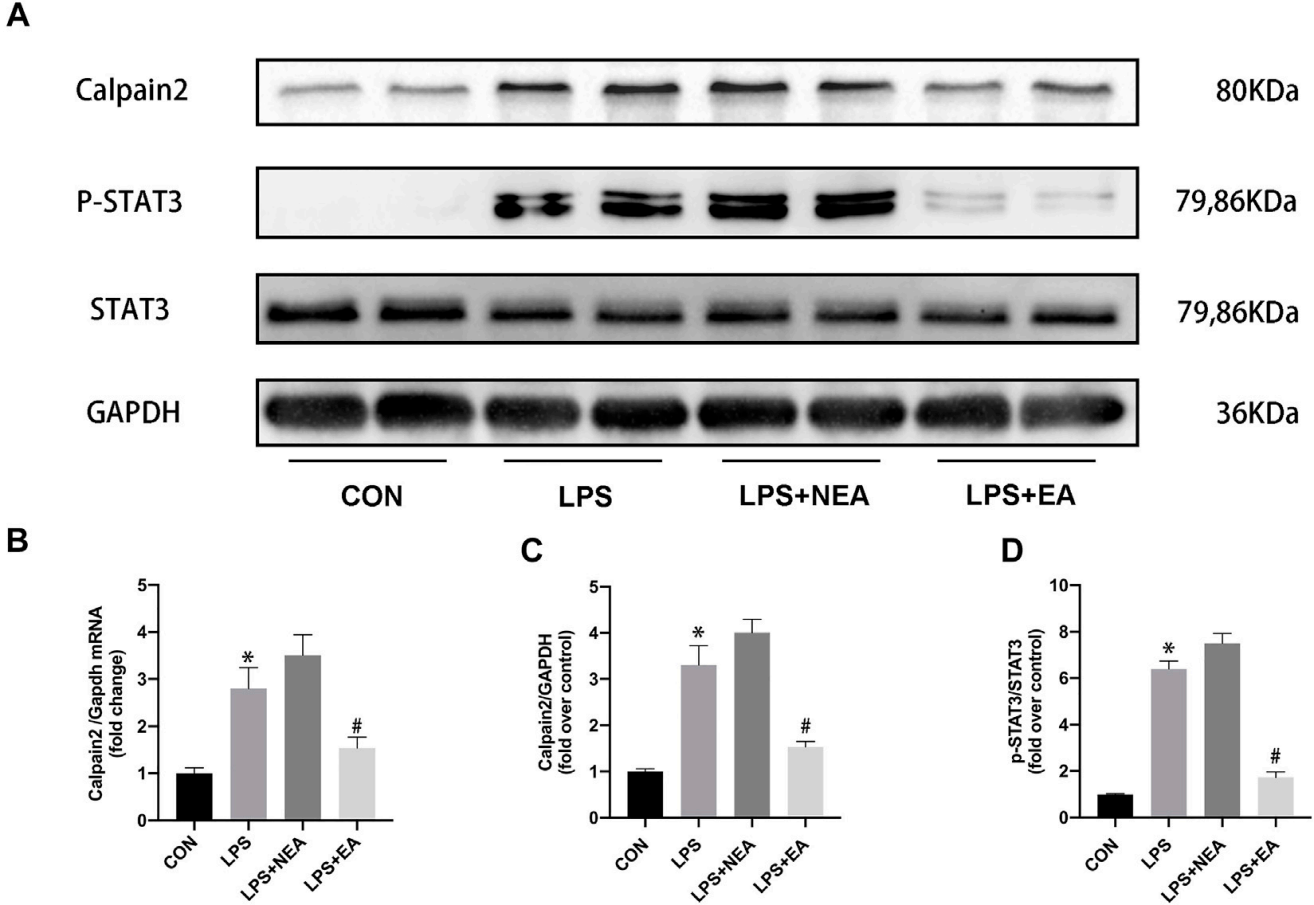
Effect of EA pre-treatment on the expression of calpain-2/STAT3. **(A)** Representative blot images of proteins related to calpain-2/p-STAT3. **(B)** mRNA expression of *Calpain-2.* **(C–D)** Quantitative analysis of calpain-2 and p-STAT3 expression. N = 4 per group. EA, electroacupuncture; NEA, EA in non-acupoint; LPS, lipopolysaccharide. *p < 0.05 vs. control group, ^#^ *p* < 0.05 vs. LPS group.

To investigate the potential molecular mechanisms underlying EA protection against septic myocardial inflammation, the expression of calpain-2 and STAT3 were assessed (Figure 3). Sepsis increased the expression of calpain-2 mRNA (Figure 3B) and pro-inflammatory protein calpain-2 (Figures 3A,C) and p-STAT3 (Figures 3A,D) compared with those in the control group. In contrast, electroacupuncture pre-treatment significantly reduced these molecules in the heart of sepsis mice.

### Inhibition of calpain-2 alleviated lipopolysaccharide-induced cardiomyocyte apoptosis and inflammation by regulating STAT3 expression

**FIGURE 4:**
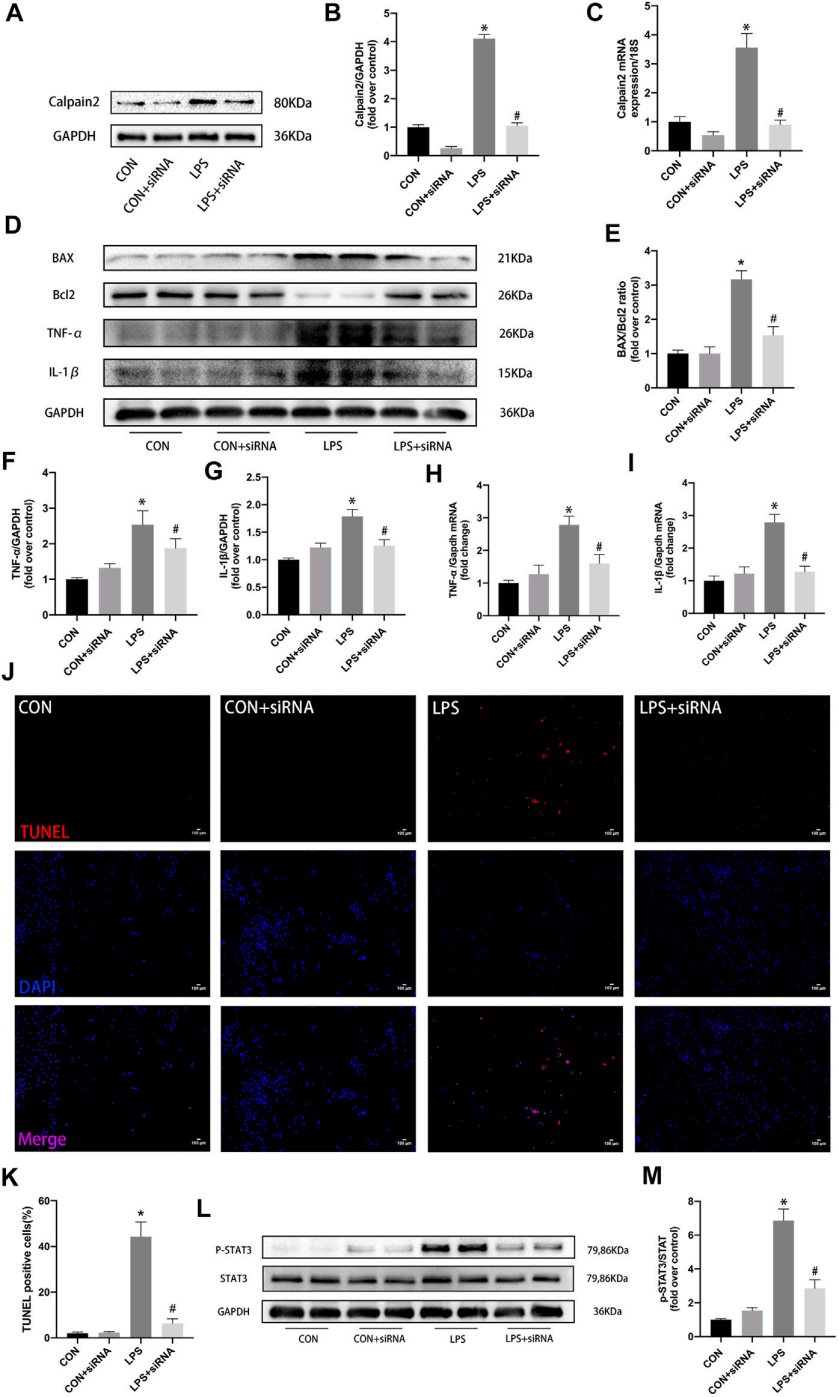
Role of calpain-2 inhibition in the expression of proinflammatory factors, apoptosis, and p-STAT3 in LPS-induced cadiomyocytes. **(A)** Representative blot images of calpian-2 using calpain-2 siRNA. **(B–C)** The quantitative analysis of protein and mRNA expression of calpain-2. **(D)** Representative images of western blots for BAX,Bcl2,TNF-α and IL-1β. **(E–G)** The quantitative analysis of protein expression of BAX\Bcl2,TNF-α and IL-1β. **(H–I)** mRNA expression of *Tnf-α* and *Il-1β* in mouse heart. **(J)** TUNEL staining, apoptosis positive cells (red), nuclei (blue). **(K)** Quantitative analysis for TUNEL postive cells. **(L)** Representative images of western blots for P-STAT3, STAT3 and GAPDH. **(M)** Quantitative results of P-STAT3 to STAT3 total protein. N = 4 per group. *p < 0.05 vs. CON group, ^#^ *p* < 0.05 vs. LPS group.

To further investigate the anti-inflammatory and anti-apoptosis roles of calpain-2 in LPS-induced cardiomyocytes, calpain-2 siRNA was used in an *in vitro* study (Figure 4). Calpian-2 siRNA effectively reduced the protein and mRNA expression of calpian-2 in neonatal cardiomyocytes (Figures 4A–C). Calpain-2 inhibition significantly reduced LPS-induced pro-inflammatory factors (TNF-α and IL-1β) in cardiomyocytes (Figures 4D,F-I). Calpian-2 siRNA treat decreased the expression of BAX and increased the expression of Bcl2 in LPS-stimulated cardiomyocytes (Figures 4D,E). Similarly, TUNEL staining showed that there were less positive cells in calpain-2 siRNA group compared with LPS treated group (Figures 4J,K). Furthermore, calpain-2 siRNA decreased p-STAT3 expression in LPS-stimulated cardiomyocytes (Figures 4L–M). These results indicate that calpian-2 inhibition has an anti-inflammatory and anti-apoptosis roles by inhibiting STAT3 activity.

### Inhibition of STAT3 enhanced anti-inflammatory response of Electroacupuncture in the lipopolysaccharide-induced mouse heart

**FIGURE 5:**
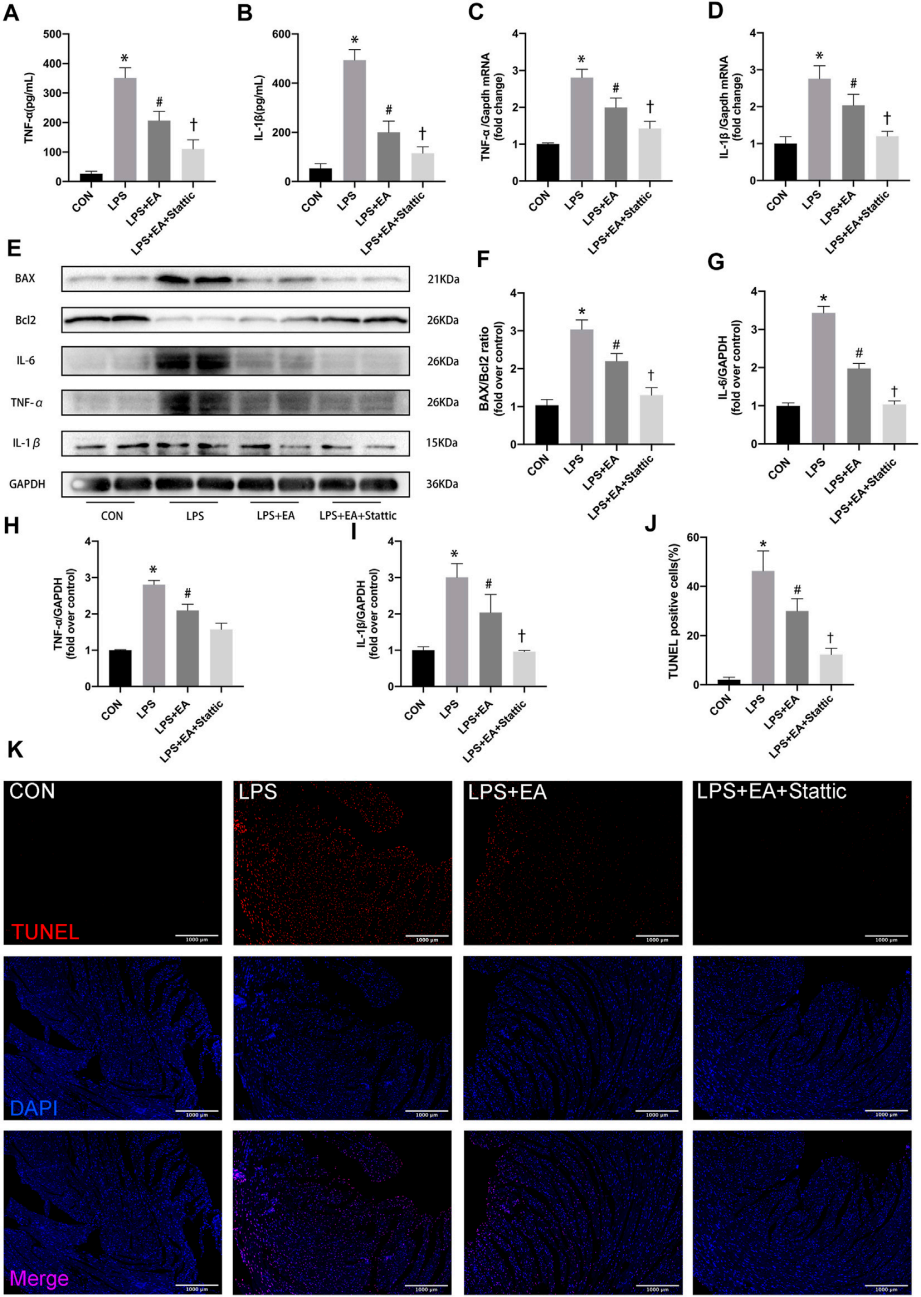
Stattic inhibited STAT3 and enhanced the anti-inflammatory and anti-apoptosis effects in EA pre-treatment sepsis mice. **(A,B)** Serum concentrations of TNF-α and IL-1β. **(C,D)** mRNA expression of *Tnf-α* and *Il-1β*. **(E)** Representative images of western blots forBAX,Bcl2, IL-6, TNF-α, IL-1β, and GAPDH. **(F)** BAX/Bcl2 ratio. **(G–I)** Quantitative results of IL-6, TNF-α, and IL-1β. **(J–K)** TUNEL staining and Quantitative analysis, apoptosis positive cells (red), nuclei (blue). N = 4 per group. EA, electroacupuncture; LPS, lipopolysaccharide. *p < 0.05 vs. control group, ^#^ *p* < 0.05 vs. LPS group,^†^ *p* < 0.05 vs. LPS + EA group.

Stattic, an STAT3 inhibitor (5 mg/kg/day, 7 days), was used to inhibit STAT3 activation to examine the STAT3-dependent anti-inflammatory and anti-apoptosis mechanisms of EA (Figure 5). The treatment with STAT3 inhibitor further enhanced the protection effect of EA pre-treatment. The expression of the LPS-induced inflammatory markers IL-1β, TNF-α and IL-6 was significantly decreased after STAT3 inhibition combined with EA pre-treatment both in serum (Figures 5A,B) and in cardiac tissue (Figures 5C–E,H,I). The expression of IL-6 (Figure 5G), TNF-α, and IL-1β was further decreased in the STAT3 inhibition group compared with that in the LPS + EA group. Similarly, the proportion of Pro-apoptotic proteins (Figures 5E,F)and TUNELpositive cells (Figures 5J,K)were further reduced compared with the acupuncture group after STAT3 inhibition. These results indicate that EA pre-treatment combined with STAT3 inhibition, more effective than single treatment, exhibited synergistic protective effects against sepsis.

## Discussion

This study demonstrated that EA pre-treatment attenuated LPS-induced cardiac dysfunction and myocardial inflammation. The underlying molecular mechanism of EA protection was found to involve the inhibition of the calpain-2/STAT3 pathway. Furthermore, the *in vitro* analysis results revealed that the inhibition of calpain-2 decreased STAT3 activity, and downregulated the pro-inflammatory pathway and reduced apoptosis in LPS-induced cardiomyocytes, indicating the importance of the calpain-2-mediated pathway in alleviating sepsis-induced heart injury.

Traditional Chinese medicine has been used to treat various diseases. Acupuncture and moxibustion (including acupuncture and electroacupuncture) has the feedback regulation effect of multiple afferent pathways. In our study, electroacupuncture was used to stimulate ST36 and PC6 Points, which was based on the principle of double acupoint matching of anti-inflammatory and heart protection. As a non-pharmacological means, EA has been shown to be a beneficial treatment strategy for several diseases including stroke (Kim et al., 2018), inflammatory pain (Xiang et al., 2019), as well as spinal cord injury (Wu et al., 2017) through clinical studies. EA is a promising method for treating cardiac diseases, such as atrial fibrillation, myocarditis, and heart failure (Sun et al., 2013; Ma et al., 2014). Moreover, previous studies have shown that EA repressed the expression of inflammatory cytokines in the hippocampus and plasma of rats with vascular dementia (Wang et al., 2020). EA at acupoint ST36 improve Th1-mediated allergic skin inflammation by restoring the Th1/Th2 balance via the suppression of Th1 differentiation. Thus, EA is a useful and promising therapeutic strategy for inflammation (Wang et al., 2017).

Mice injected with LPS intraperitoneally represent a well-known animal model of systemic inflammation and cardiac dysfunction. Here, LPS injection decreased the EF and FS, consistent with the findings of previous studies (Liu L et al., 2021; Mou et al., 2021; Qiao et al., 2021). We observed that EA pre-treatment for seven consecutive days increased the EF, FS, and E/A ratio but suppressed the activities of calpain and reduced apoptosis and pro-inflammatory factors in septic mouse hearts.

These results showed that EA pre-treatment attenuated LPS-induced cardiac dysfunction and myocardial apoptosis. We focused on the anti-inflammatory mechanism of acupuncture in septic cardiomyopathy. Accumulating evidence indicates that pro-inflammatory factors are considerably activated in the LPS-injured myocardium (Okuhara et al., 2017; Qiao et al., 2021). Here, the expression of cytokines (TNF-α and IL-1β) was upregulated in both serum and cardiac tissues in LPS-induced mouse. In addition, the TNF-α,IL-1βand IL-6 levels were increased in septic mouse hearts; however, EA pre-treatment prevented the response mediated by these pro-inflammatory cytokines. These results show that EA pre-treatment attenuated the LPS-induced cardiac inflammatory response*.*

Growing evidence suggests that the calpain/calpastatin system plays a critical role in the association between sepsis and multi-organ system dysfunction (Huang et al., 2021; Rurik et al., 2021). Among the calpain family members, calpain-1 and calpain-2 have been studied extensively. Both calpain-1 and calpain-2 are tightly regulated by the intracellular concentration of free Ca^2+^and the endogenous inhibitor calpastatin (Goll et al., 2003). Peng et al. demonstrated that the administration of LPS activates both NADPH/NADH oxidase and calpain-1 in cardiomyocytes, whereas NADPH oxidase inhibition abrogates calpain-1 overactivation (Huang et al., 2021). Moreover, the administration of calpain inhibitor (calpain inhibitor-Ш or PD150606) prevents LPS-induced degradation of myocardial Hsp90/p-Akt and its expression in cardiomyocytes, in addition to inhibiting myocardial caspase-3 activation and apoptosis (Li et al., 2013). Similarly, overexpression of calpastatin inhibits calpain activation and attenuates myocardial dysfunction during endotoxaemia (Li et al., 2009). In our study, the expression of calpain-2, which was decreased by EA pre-treatment, increased significantly in LPS-induced mouse hearts may contribute to cardiomyocyte apoptosis. Indeed, we further confirmed the protective effect of calpain-2 inhibition on the inflammatory response and apoptosis in LPS-stimulated cardiomyocytes. In addition, inhibition of calpain-2 can also reduce the expression of p-STAT3. However, how calpain-2 directly regulates p-STAT3 still needs further research.

The key role of STAT3 signalling in the pro-inflammatory response in septic cardiomyopathy has been illustrated in previous studies (Jin et al., 2017); STAT3 may contribute to the development of systemic inflammation in sepsis. Consistently, our results showed that EA reduced p-STAT3 expression by inhibiting calpain-2 (Chen et al., 2017). More importantly, our results showed that pharmacological inhibition of STAT3 activity could enhance the anti-inflammatory and anti-apoptotic effects of electroacupuncture.

Electroacupuncture may induce remote ischemic conditioning to attenuate septic cardiomyopathy, improve cardiac output, protect systemic organs, and improve mortality in a lipopolysaccharide-induced sepsis model (Honda et al., 2019). Similar to EA,transcutaneous electrical nerve stimulation induced marked cardioprotection with significantly reduced infarct size after ischemia/reperfusion (I/R) injury (Merlocco et al., 2014). Dialysate from EA animals led to significant reduction in infarct size and improved functional recovery after I/R injury; The degree of cardioprotection was no different to that seen in animals randomized to receive remote preconditioning using transient limb ischemia (Redington et al., 2013). Therefore, neuronal and humoral mediators transfer the EA induced protective signal from the periphery to the heart (Kleinbongard et al., 2017). One study found that electroacupuncture stimulation (ES) drives sympathetic pathways in somatotopy- and intensity-dependent manners (Liu et al., 2020). Low-intensity ES (0.5 mA) at hindlimb regions (Zusanli) drives the vagal-adrenal axis, producing anti-inflammatory effects that depend on NPY + adrenal chromaffin cells, also decreased the TNF-αand improved survival. High-intensity ES (3 mA)at the abdomen activates NPY + splenic noradrenergic neurons via the spinalsympathetic axis. However, high-intensity pre-electroacupuncture stimulation at the abdomen was beneficial to the survival of sepsis mice, but post-treatment decreased the survivals (Liu et al., 2020). In our study, also confirmed by other researchers (Liu S et al., 2021), low-intensity electroacupuncture pretreatment of Neiguan and Zusanli may reduce the proinflammatory effect of hyperactivated calpain-2 and p-STAT3 through the vagosplenic axis (Lieder et al., 2018).

However, some researchers confirmed that remote ischemic preconditioning reduced the size of myocardial infarction and was associated with increased phosphorylation of STAT3s in pigs (Kleinbongard et al., 2018; Heusch, 2019; Heusch, 2020). 39sahnchuConversely, other studies have also pointed out STAT3 drives cardiac hypertrophy and heart failure by impairing mitochondrial bioenergetics (Zhuang et al., 2022), and STAT3 induces cardiac fibroblast activation and cardiac fibrosis (Su et al., 2017; Bao et al., 2020). The protective effects of remote ischemic conditioning and its associated STAT3 activation are through the vagosplenic axis (Lieder et al., 2018; Heusch, 2019; Liu S et al., 2021) Therefore, the difference in results may be that acute STAT three activation as in remote ischemic conditioning may be protective but chronic STAT3 activation as in sepsis may be deleterious. In this study, STAT3 activation may be related to excessive inflammatory response in the process of LPS induced cardiac inflammation and early stage of apoptosis, while STAT3 returned to normal level with the EA preconditioning.

The disadvantage of this study is that it has not studied whether stimulation of Neiguan alone has the same effect, because Zusanli has been proven to be effective. In addition, it is not known whether the EA post-treatment of Zusanli and Neiguan after LPS injection can also play a protective role through calpain-2/p-STAT3 axis. Importantly, we confirmed that electroacupuncture combined with STAT3 inhibition was more effective in protecting sepsis cardiomyopathy than electroacupuncture alone. Unlike animals, EA treatment will cause stress, EA post-treatment in clinical sepsis may have the same effect as pre-treatment for protecting heart. Because calpian-2 can regulate p-STAT3, further study on the therapeutic effect of electroacupuncture combined with calpain may be more beneficial to clinical application *in vivo* experiments.

## Conclusion

In summary, to the best of our knowledge, this is the first study to report that EA pre-treatment downregulates calpain-2 expression, thereby inhibiting the phosphorylation of STAT3 and improving cardiac function during sepsis. Elucidating the role of calpain-2 in the pathological process of sepsis-induced cardiac inflammation will be required to better understand the effect of EA protection, which may not only help understand the molecular mechanism of EA but also have potential clinical value. This study also verify the multi-channel feedback regulation law of electroacupuncture, which not only improves the systemic inflammation caused by sepsis, but also reduces the visceral inflammatory response. Obviously, multiple combinations of acupuncture points and stimulation points will have more value in future research and clinical application.

## Data Availability

The original contributions presented in the study are included in the article/Supplementary Material, further inquiries can be directed to the corresponding authors.
