# Supplementary figures and images for: Electroacupuncture pre-treatment alleviates sepsis-induced cardiac inflammation and dysfunction by inhibiting the calpain-2/STAT3 pathway
Source: Front Physiol. 2022 Sep 7;13:961909. doi: 10.3389/fphys.2022.961909 (PMC9489935; doi:10.3389/fphys.2022.961909)

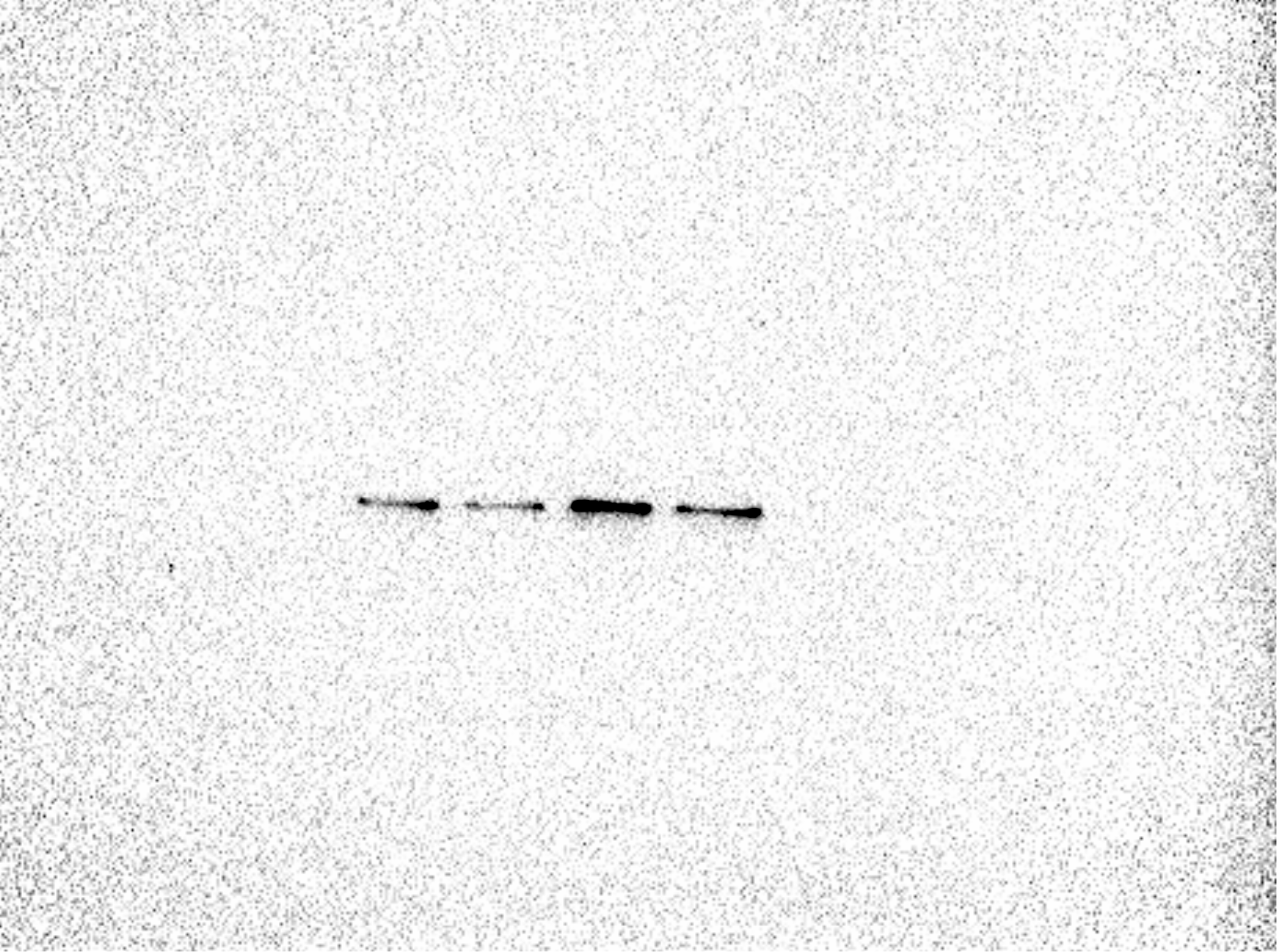

Supplement: Supplementary file 1 [file DataSheet3.ZIP › fig4 A-WB1/CALPAIN2.tif]

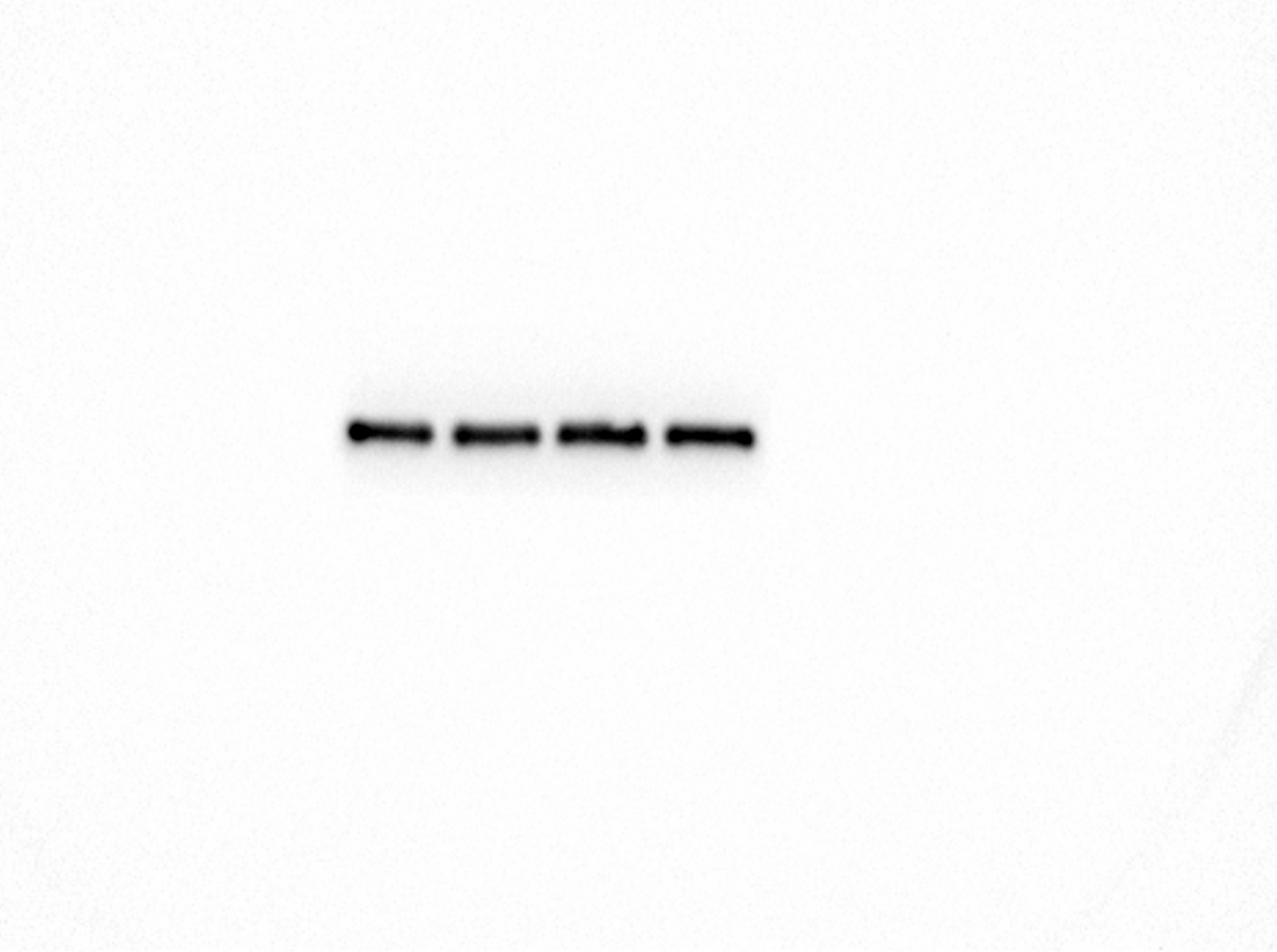

Supplement: Supplementary file 1 [file DataSheet3.ZIP › fig4 A-WB1/GAPDH╡─╕▒▒╛.tif]

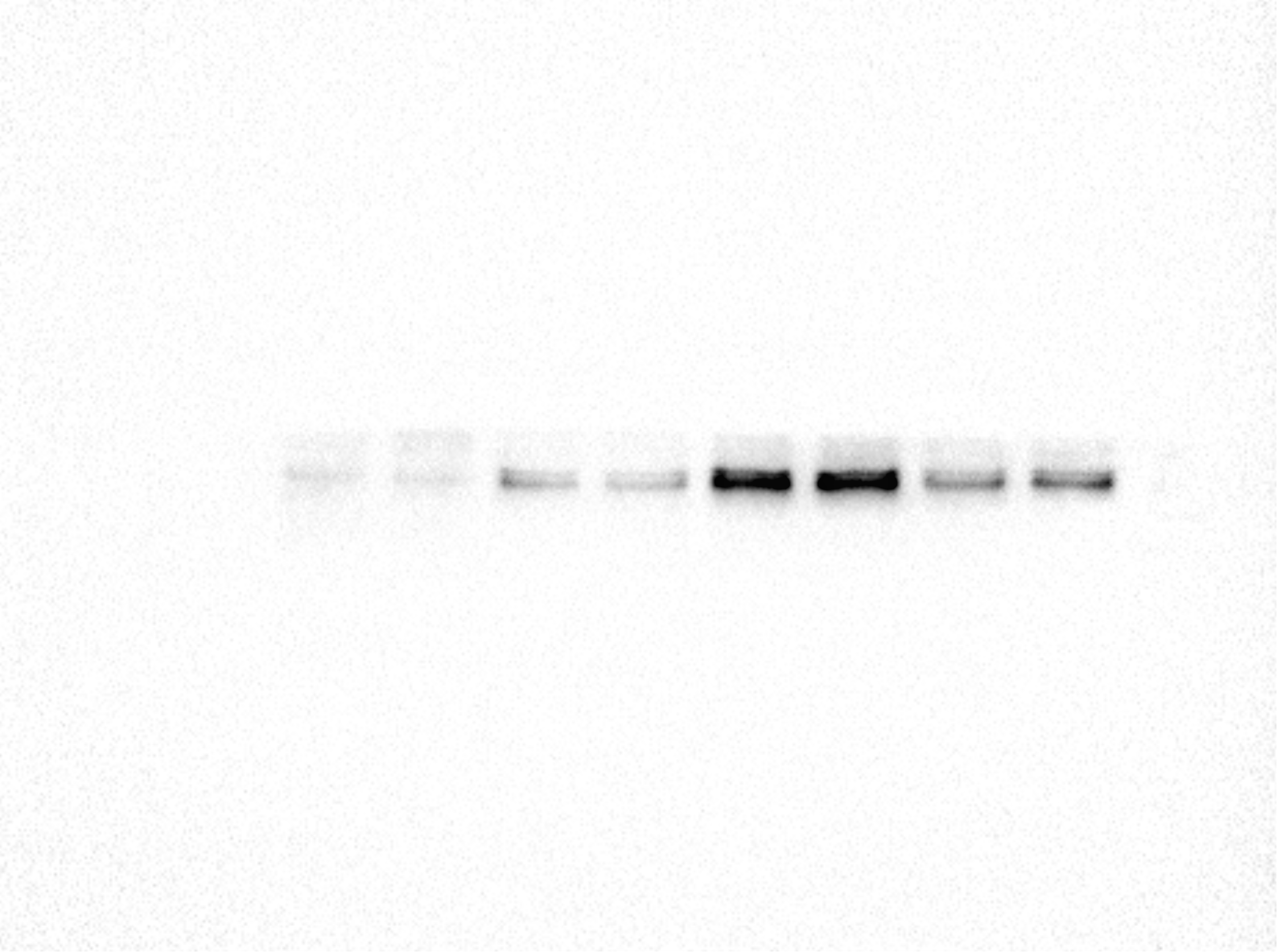

Supplement: Supplementary file 3 [file DataSheet2.ZIP › WB/FIG4-P-STAT3.tif]

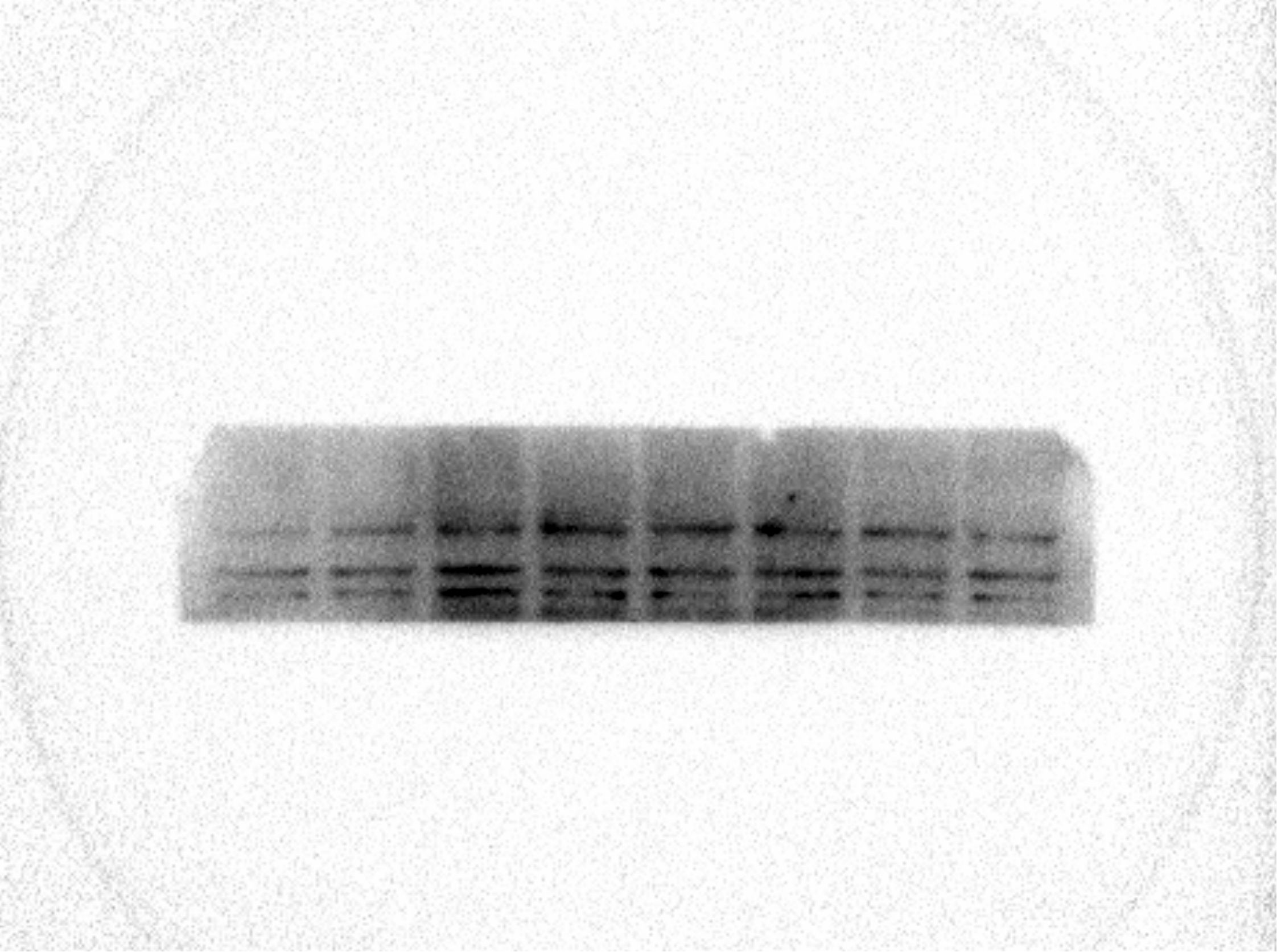

Supplement: Supplementary file 3 [file DataSheet2.ZIP › WB/FIG2-IL-1beta.tif]

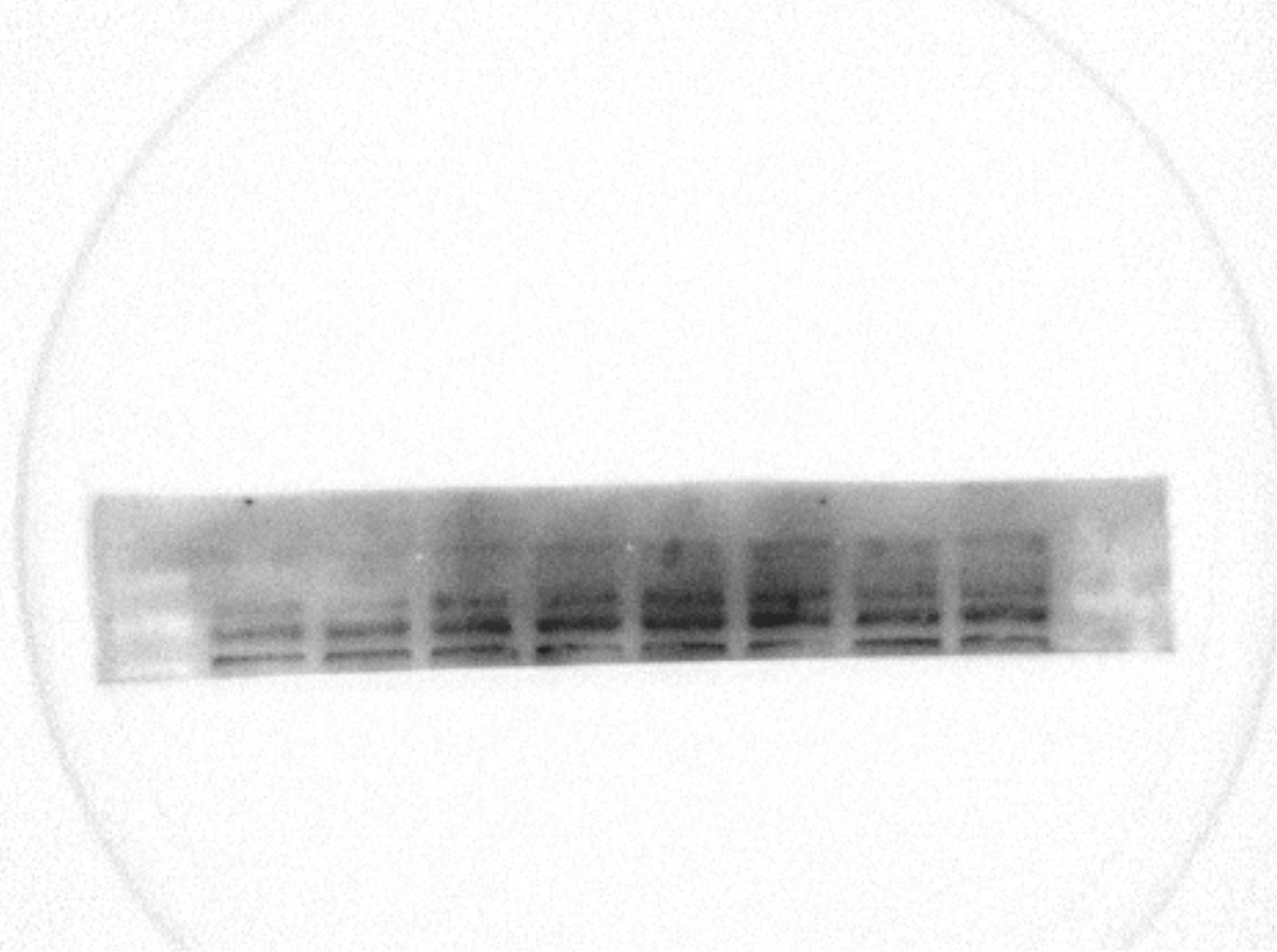

Supplement: Supplementary file 3 [file DataSheet2.ZIP › WB/FIG2-TNF.tif]

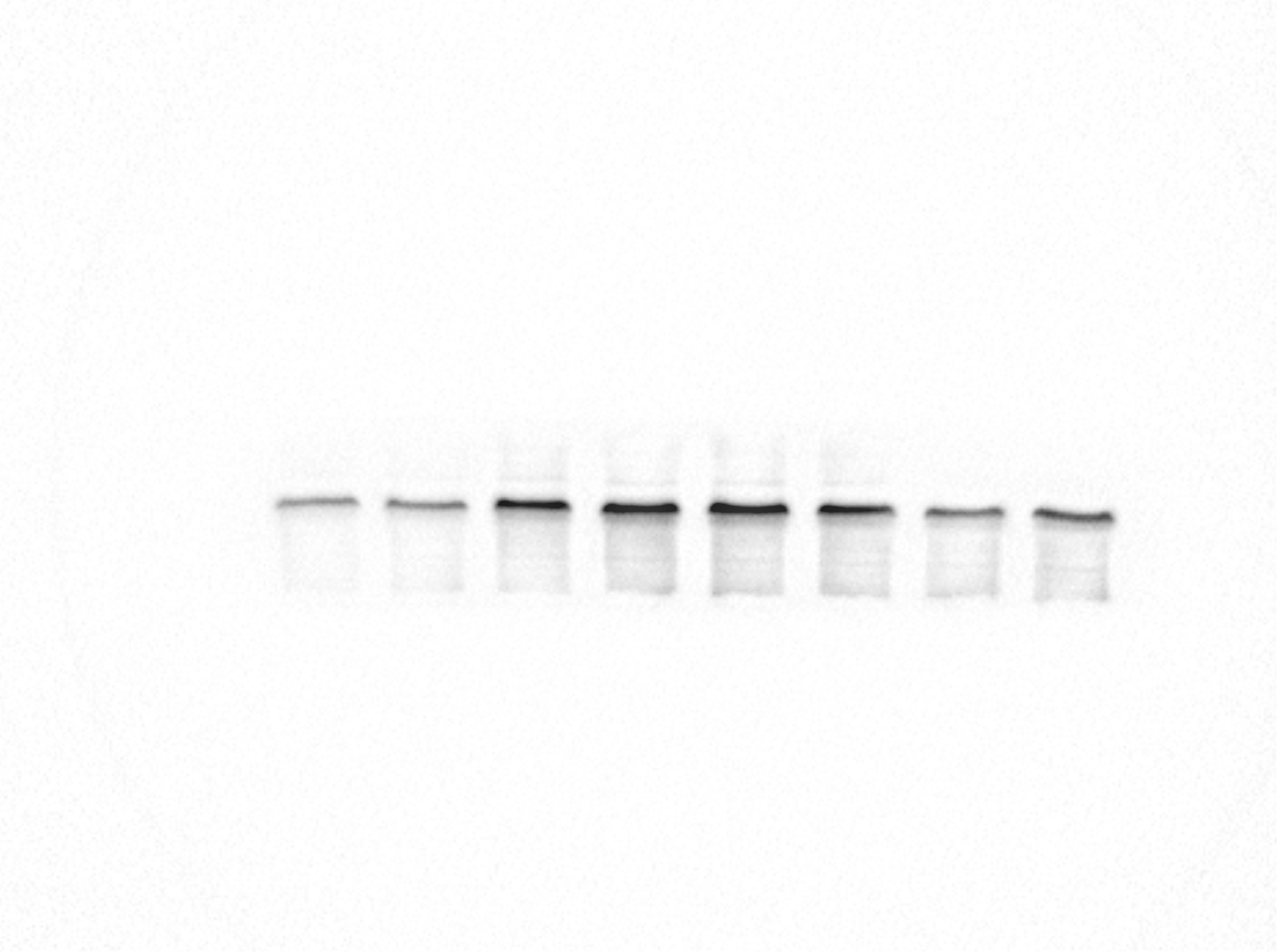

Supplement: Supplementary file 3 [file DataSheet2.ZIP › WB/FIG3-CALPAIN2.tif]

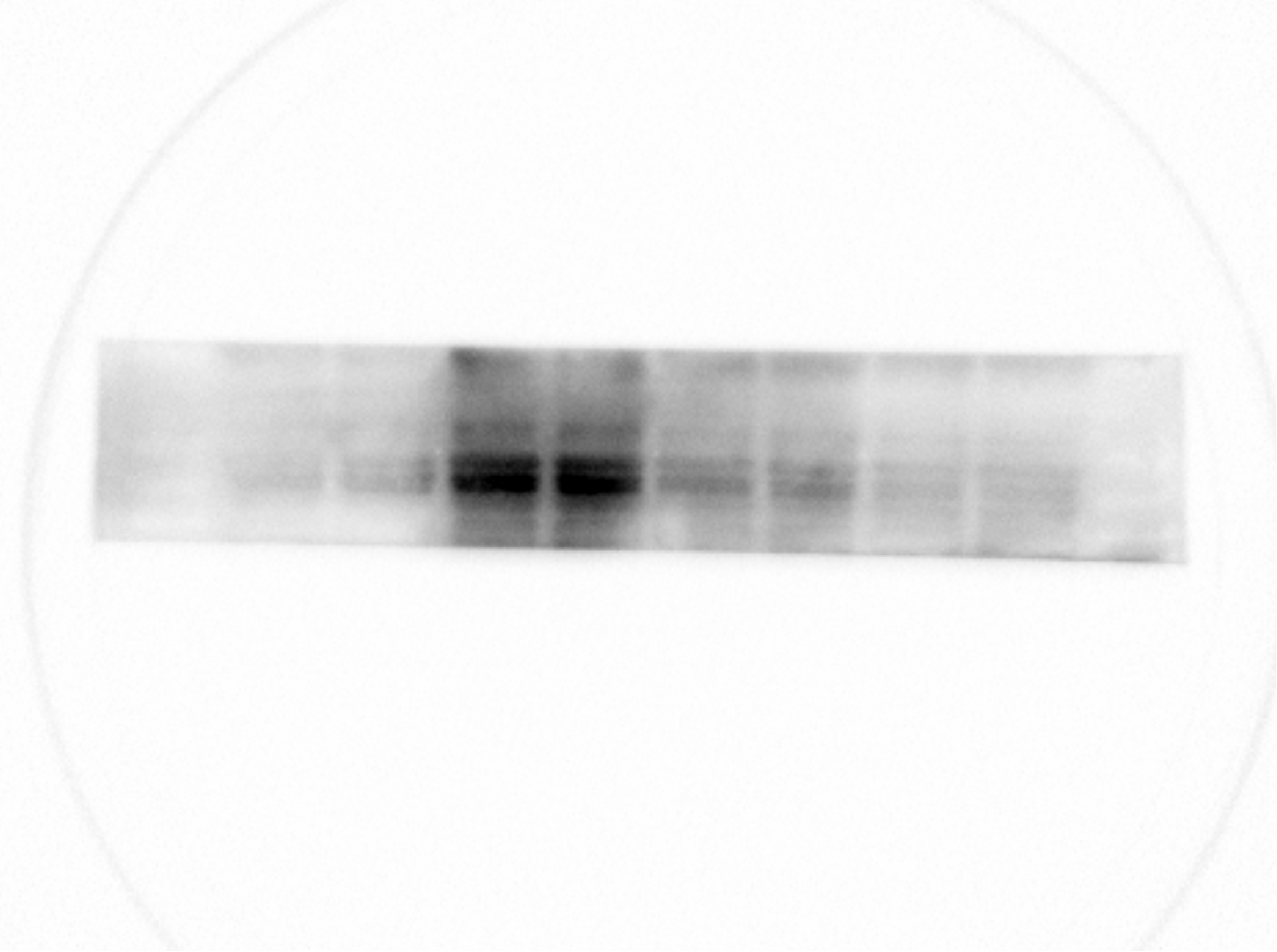

Supplement: Supplementary file 3 [file DataSheet2.ZIP › WB/FIG5-IL-6.tif]

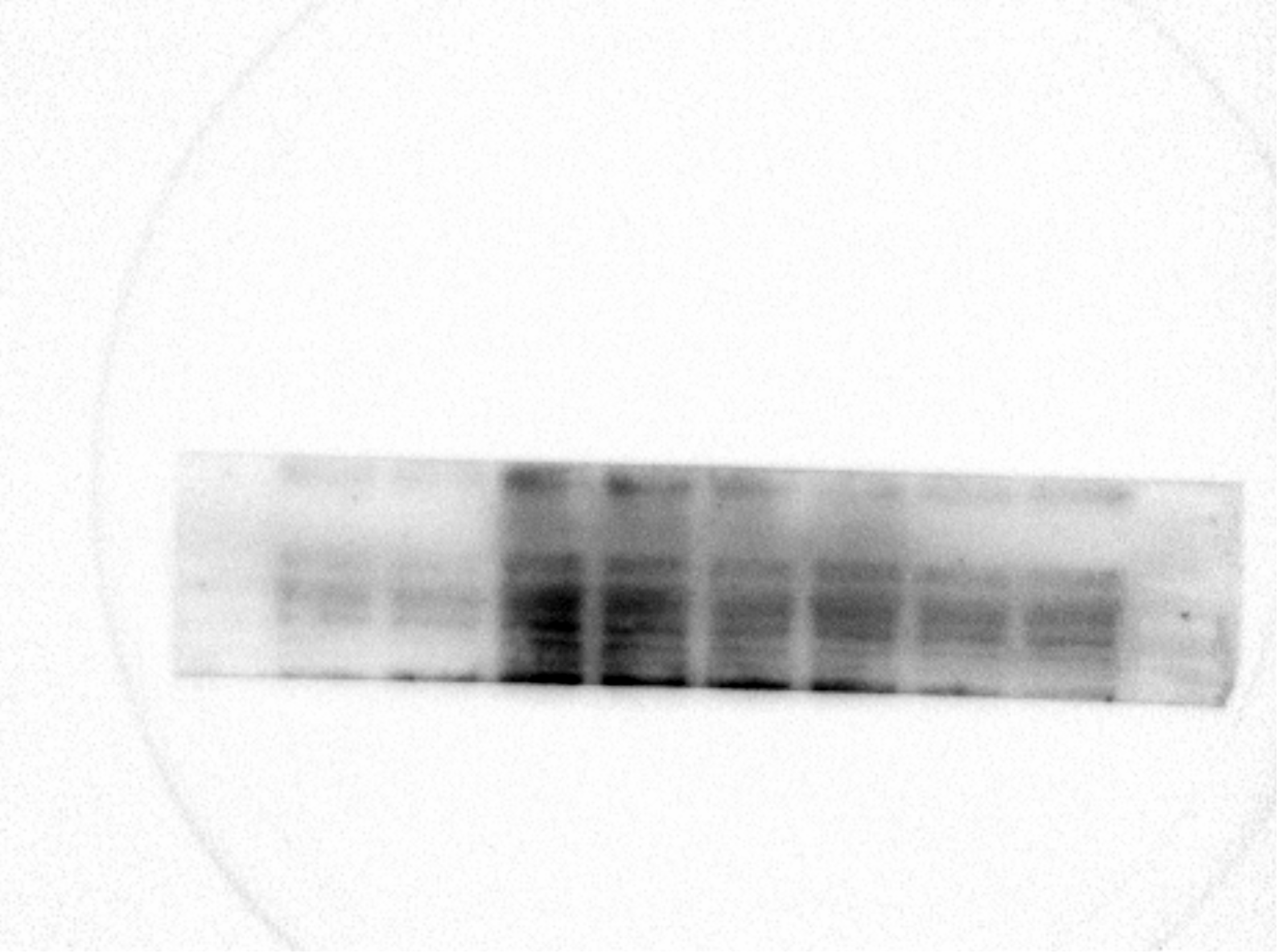

Supplement: Supplementary file 3 [file DataSheet2.ZIP › WB/FIG5-TNF.tif]

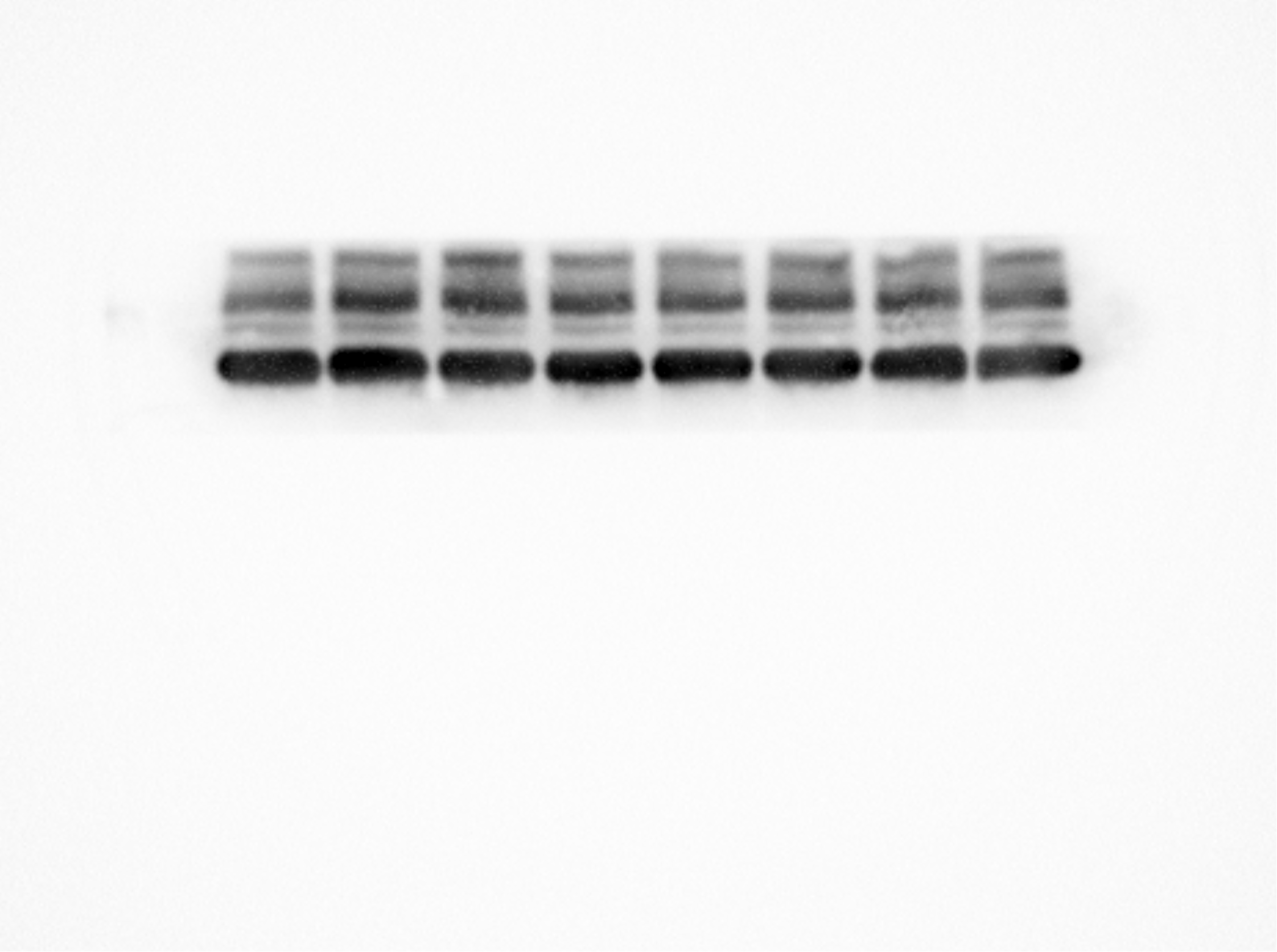

Supplement: Supplementary file 3 [file DataSheet2.ZIP › WB/FIG3-GAPDH.tif]

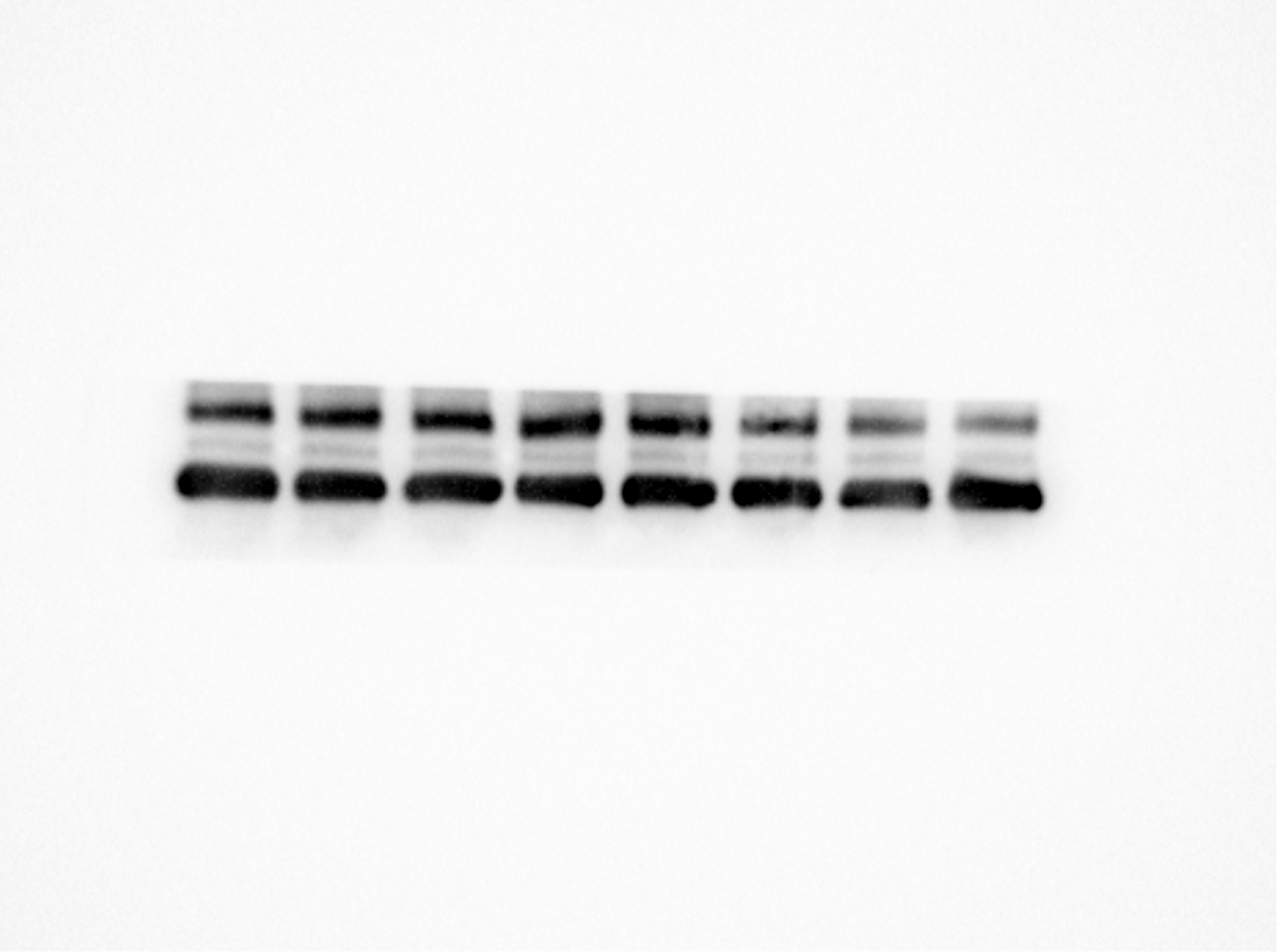

Supplement: Supplementary file 3 [file DataSheet2.ZIP › WB/FIG4-GAPDH-2.tif]

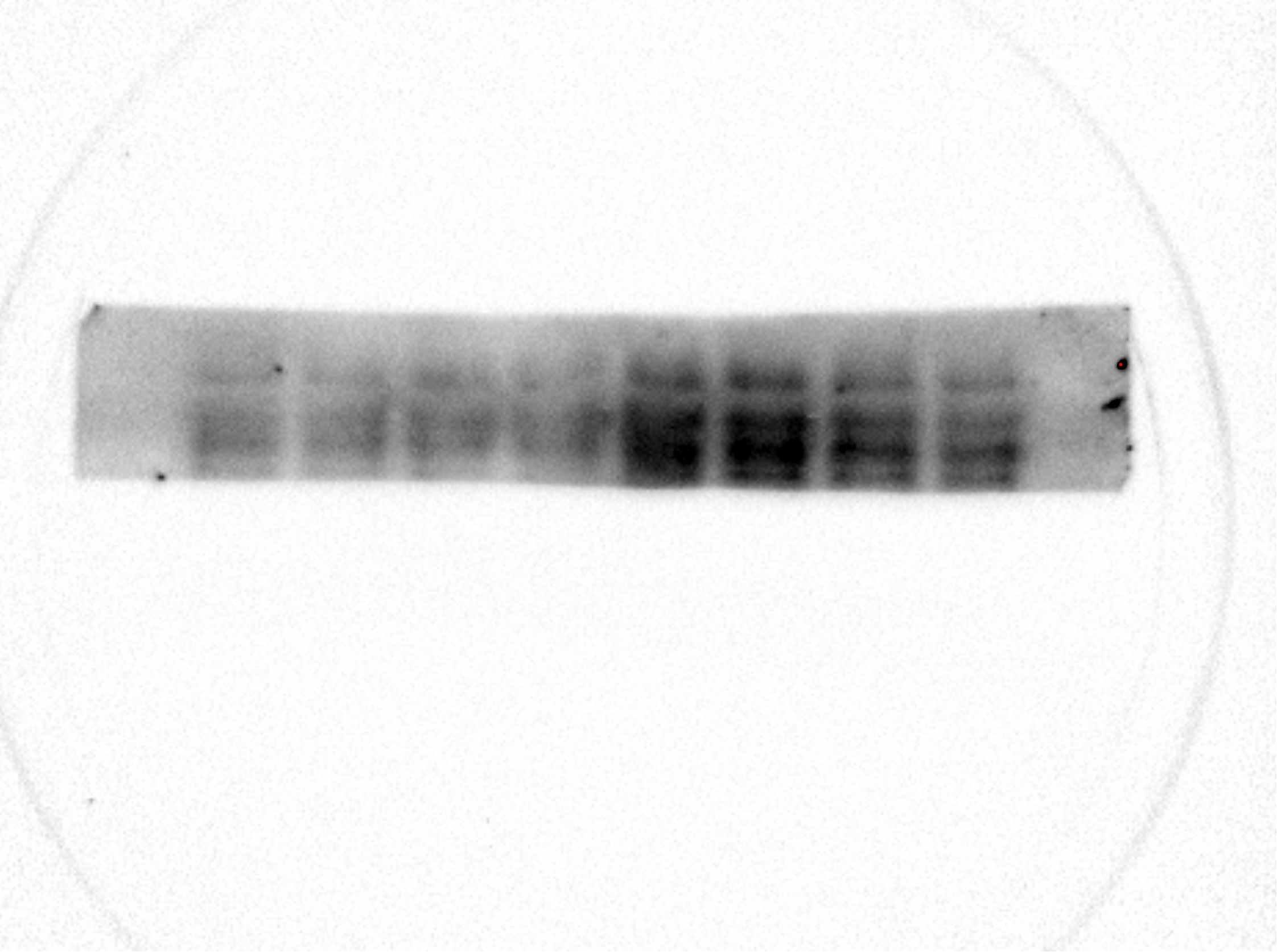

Supplement: Supplementary file 3 [file DataSheet2.ZIP › WB/FIG4-TNF.tif]

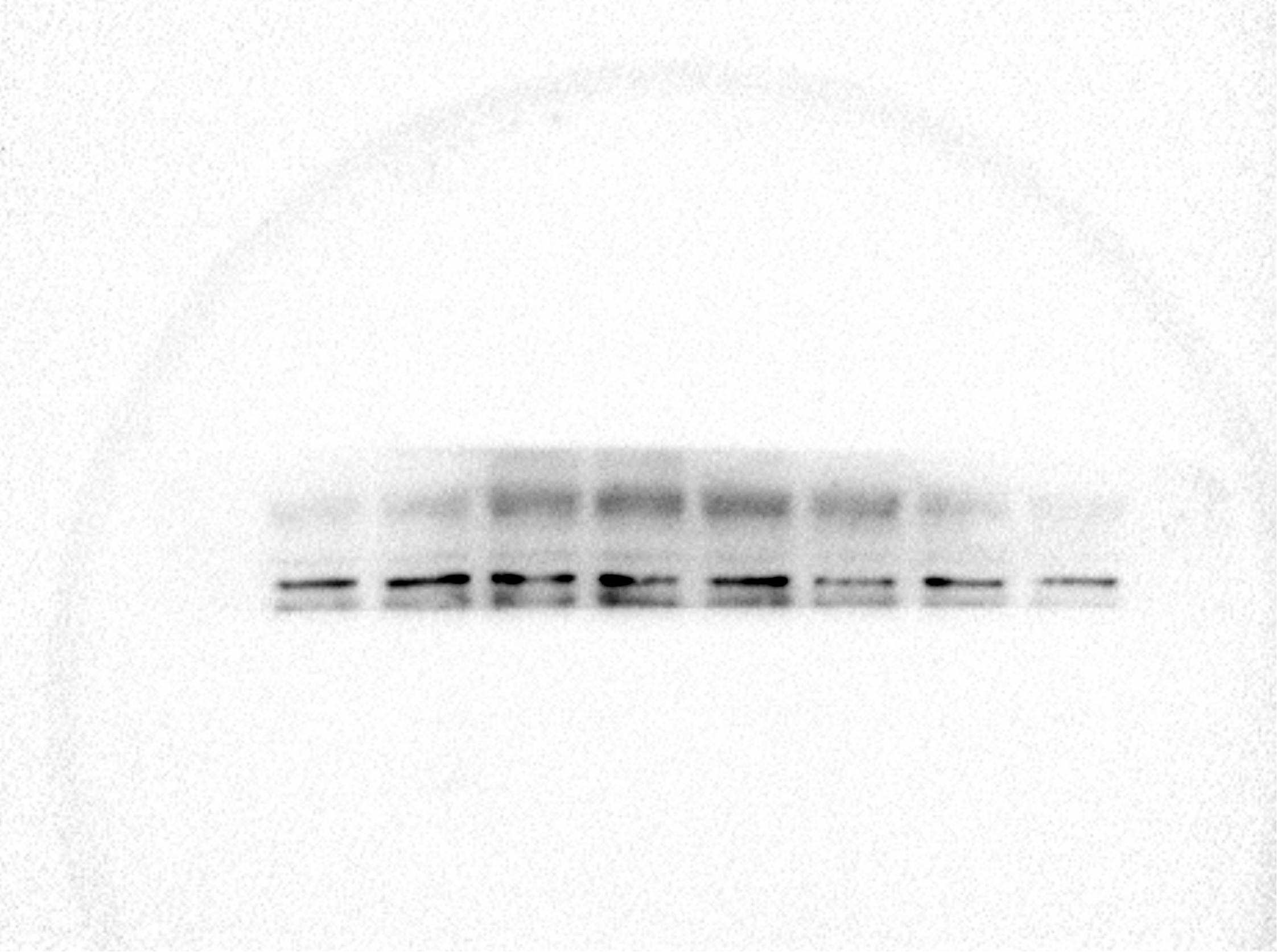

Supplement: Supplementary file 3 [file DataSheet2.ZIP › WB/FIG5-IL-1B.tif]

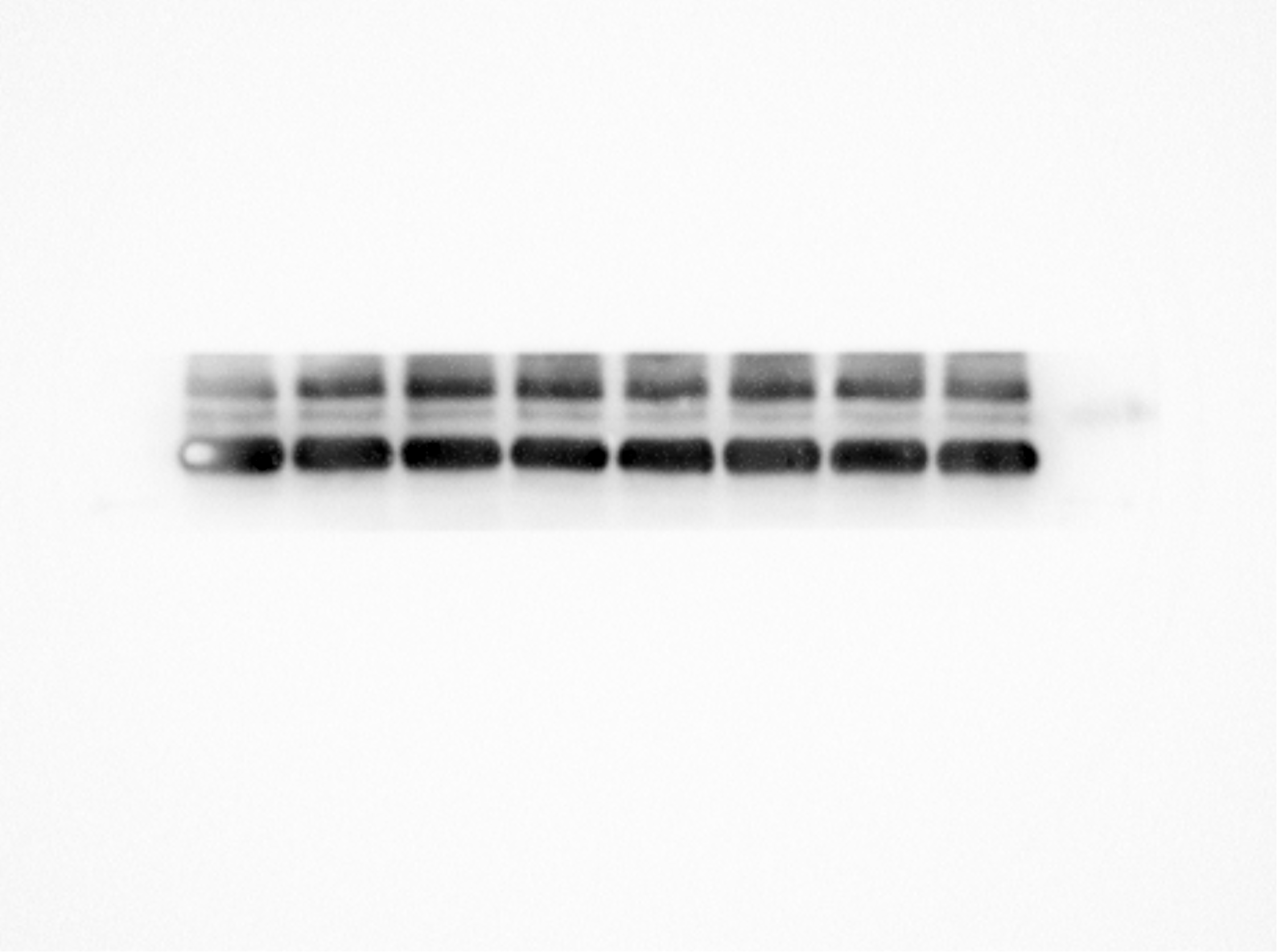

Supplement: Supplementary file 3 [file DataSheet2.ZIP › WB/FIG5-GAPDH.tif]

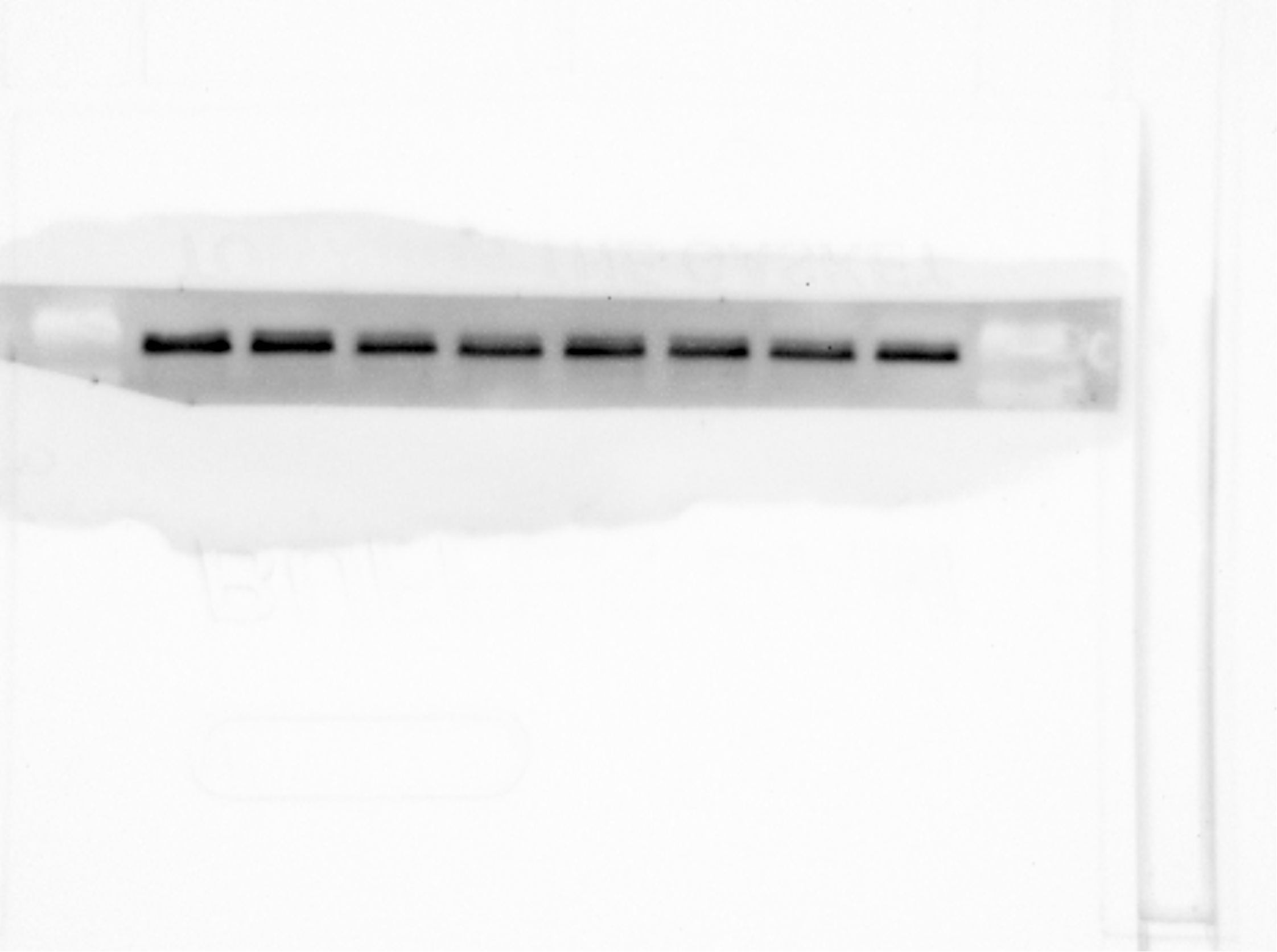

Supplement: Supplementary file 3 [file DataSheet2.ZIP › WB/FIG3-STAT3.tif]

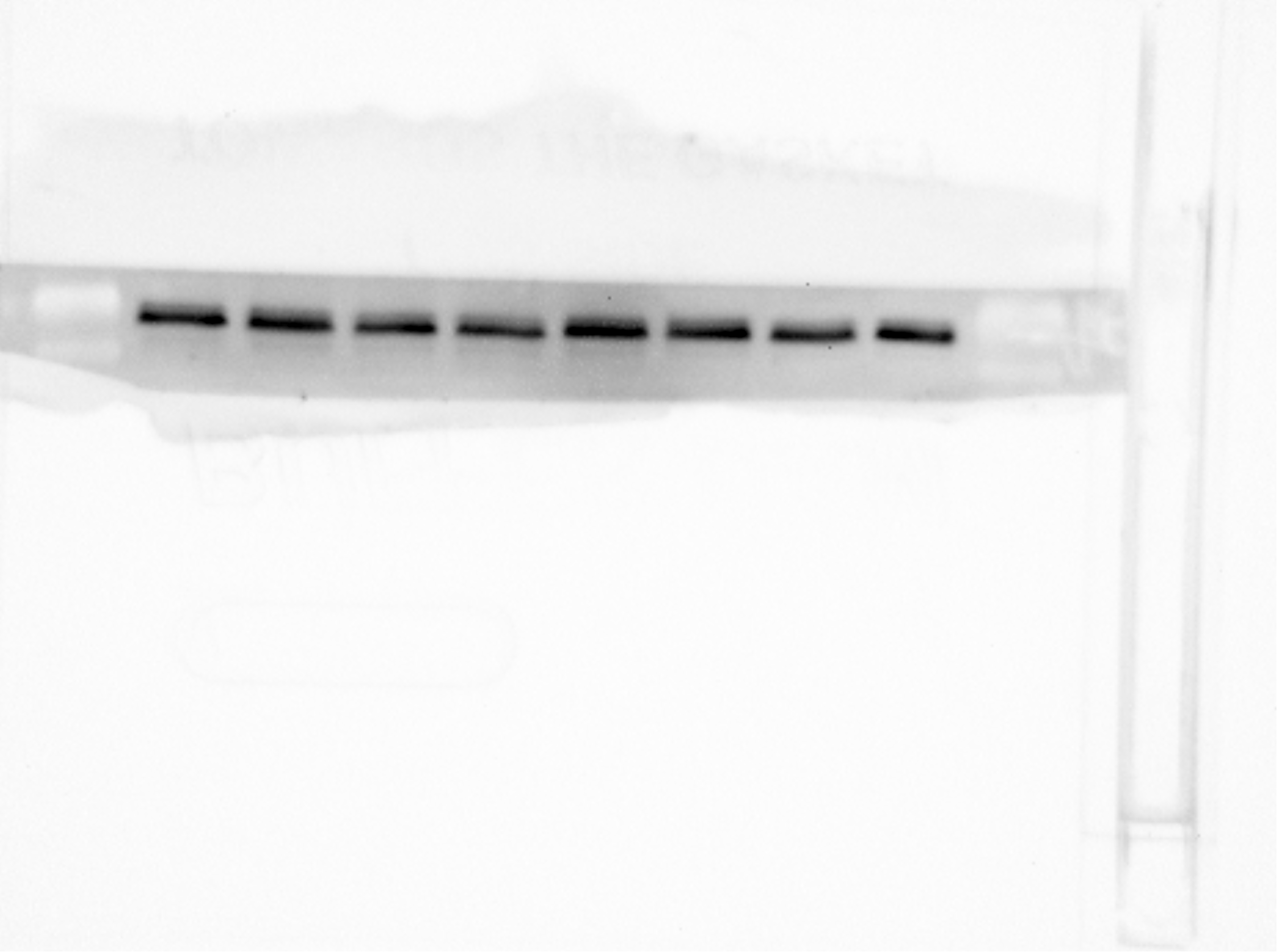

Supplement: Supplementary file 3 [file DataSheet2.ZIP › WB/FIG4-STAT3.tif]

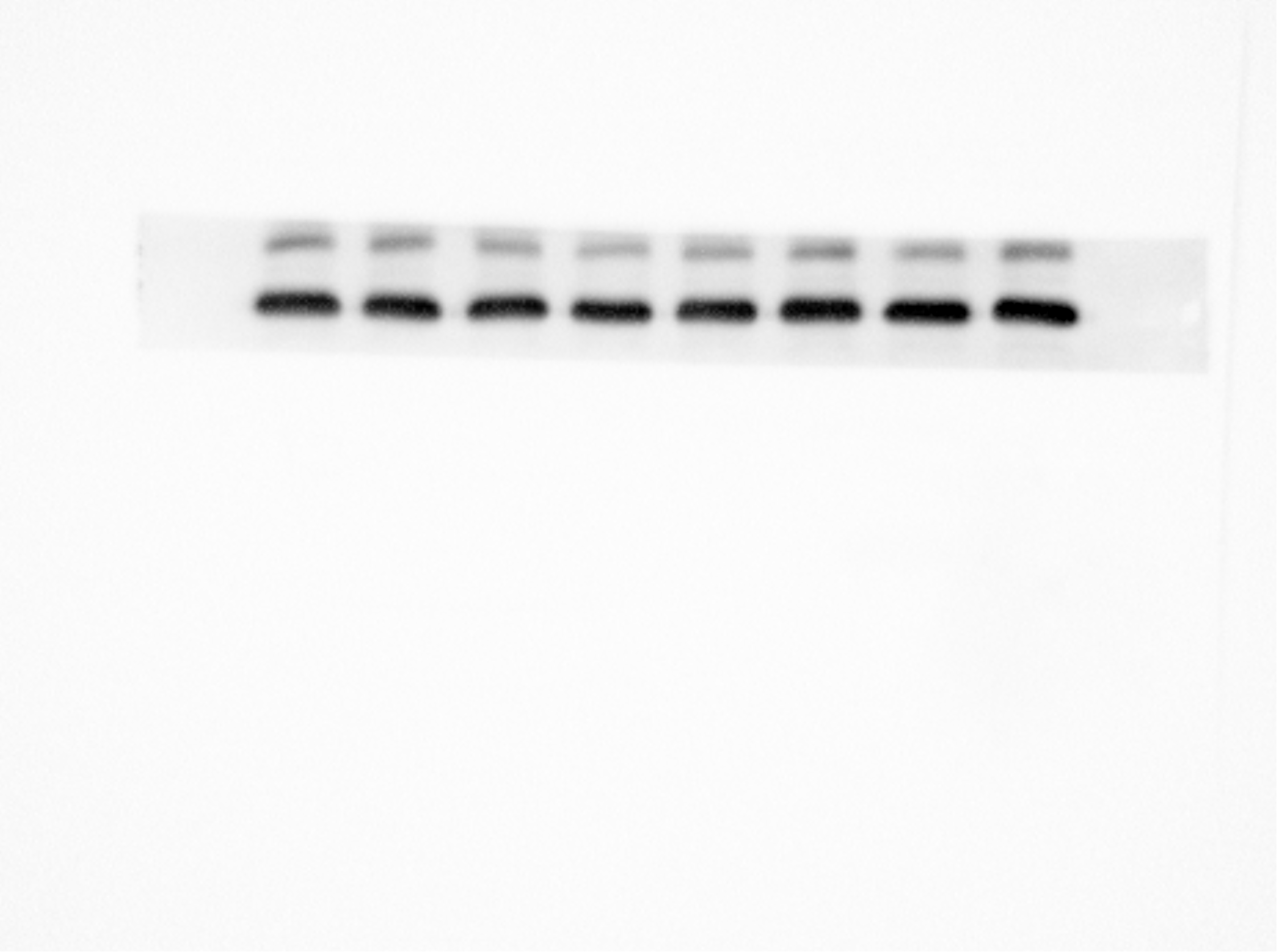

Supplement: Supplementary file 3 [file DataSheet2.ZIP › WB/FIG2-GAPDH.tif]

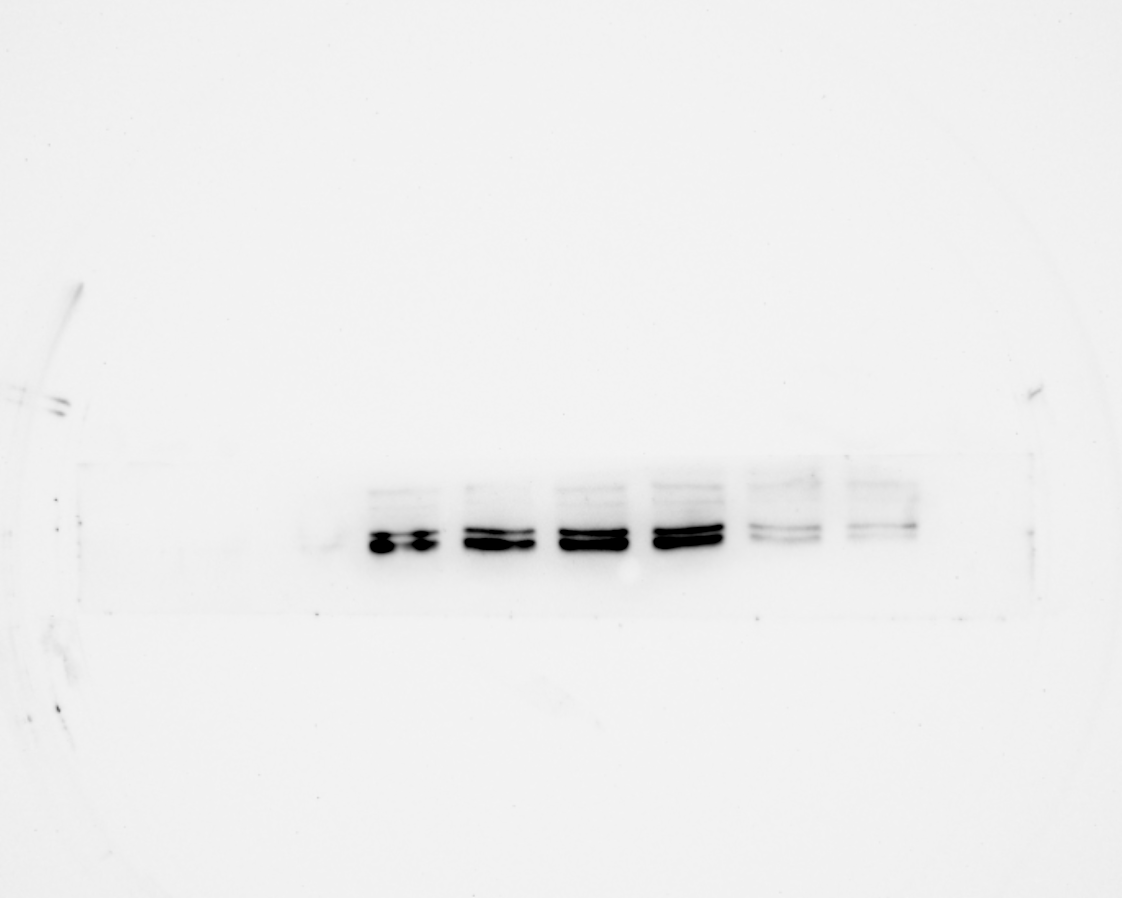

Supplement: Supplementary file 3 [file DataSheet2.ZIP › WB/FIG3-P-STAT3.tif]

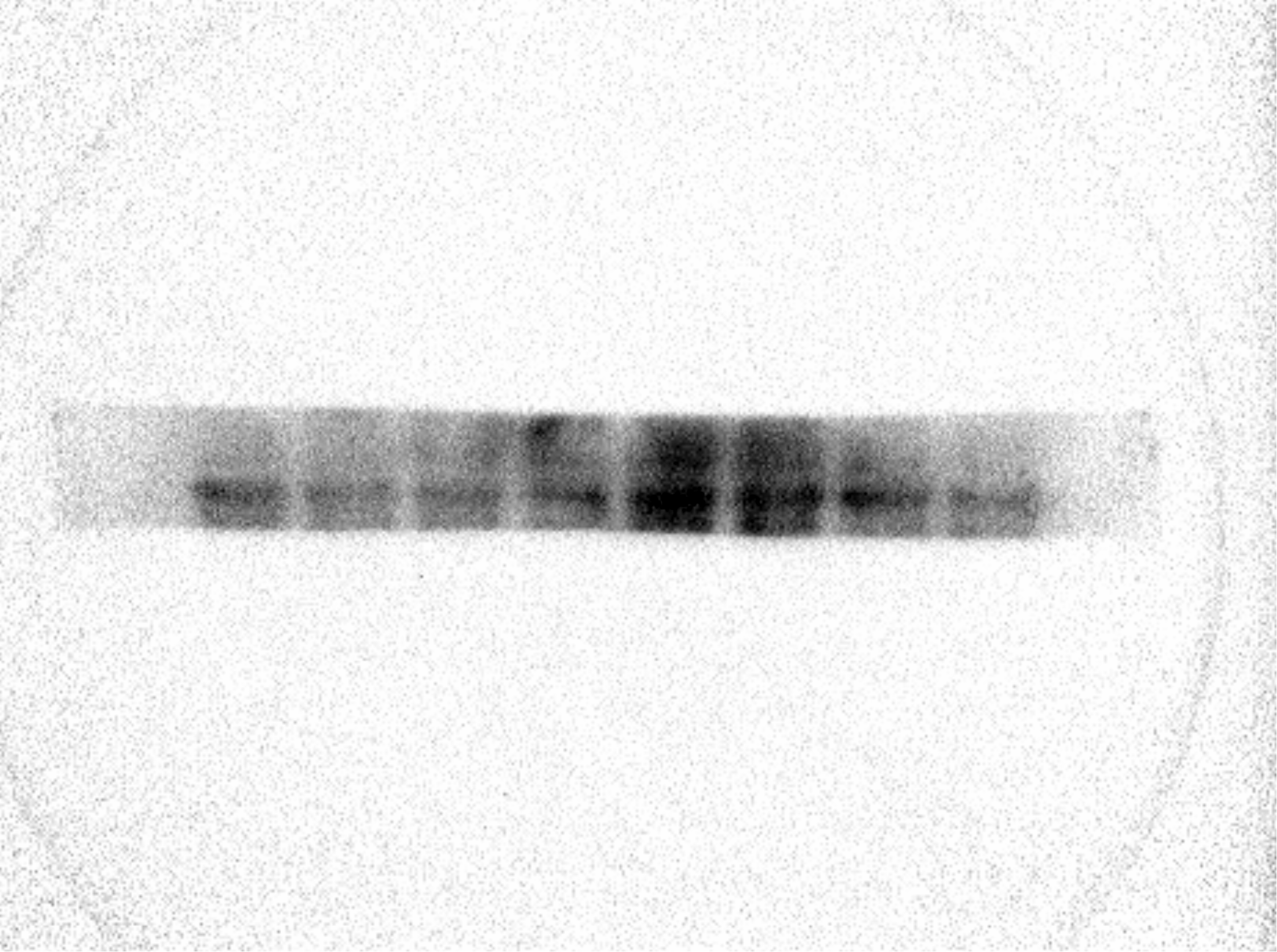

Supplement: Supplementary file 3 [file DataSheet2.ZIP › WB/FIG4-IL-1B.tif]

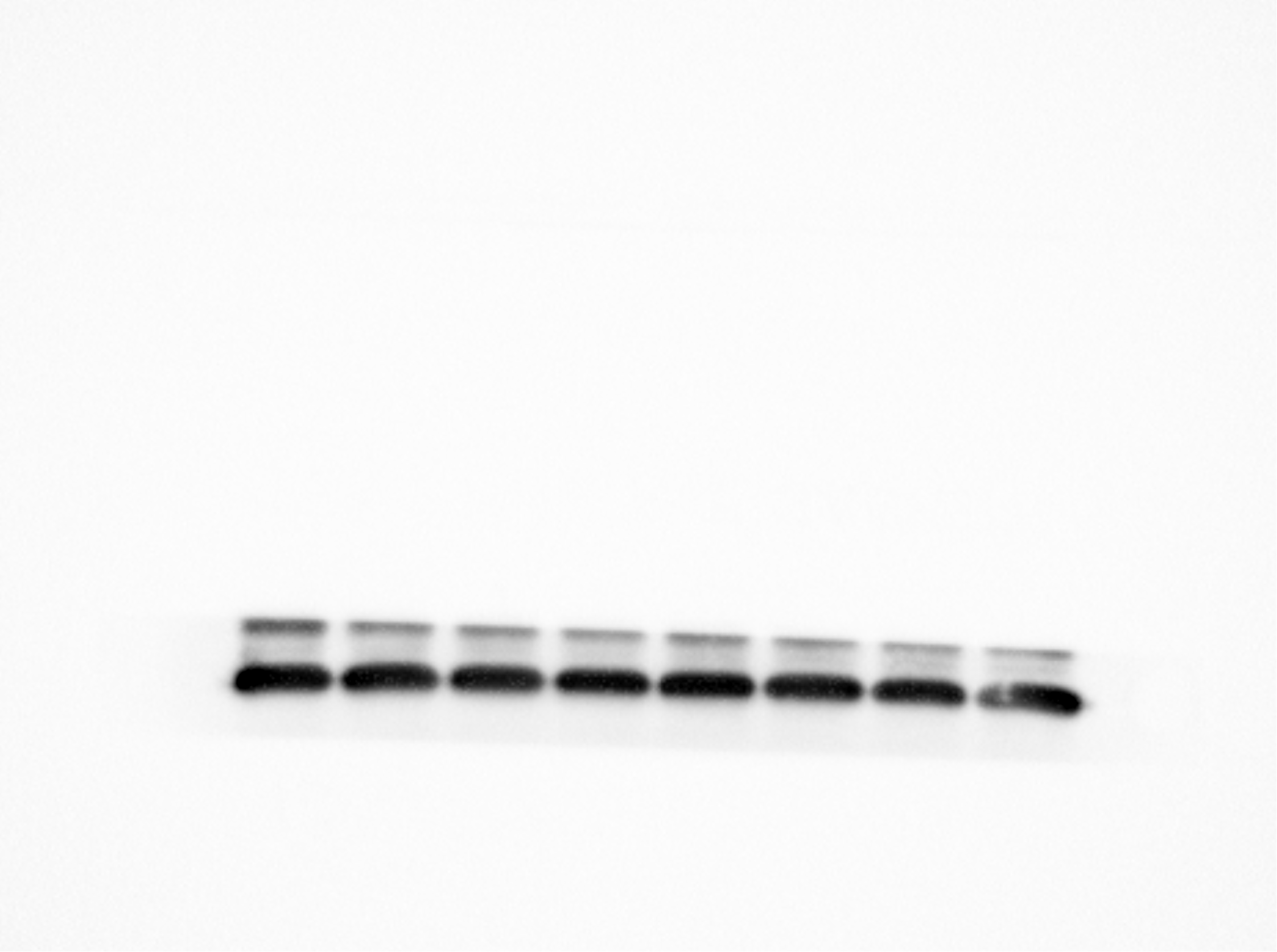

Supplement: Supplementary file 3 [file DataSheet2.ZIP › WB/FIG4-GAPDH.tif]

our data sharing on Jianguoyun ：

<https://www.jianguoyun.com/c/sd/155de74/39b9c4da1354c4a6>


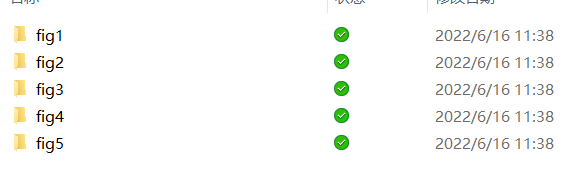

Supplement: Supplementary file 4 [file DataSheet1.DOCX]
