# Supplementary material for: Electroacupuncture pre-treatment alleviates sepsis-induced cardiac inflammation and dysfunction by inhibiting the calpain-2/STAT3 pathway
Source: Front Physiol. 2022 Sep 7;13:961909. doi: 10.3389/fphys.2022.961909 (PMC9489935; doi:10.3389/fphys.2022.961909)
Supplement: Supplementary file 2 [file Presentation1.PPTX]

## Slide 1
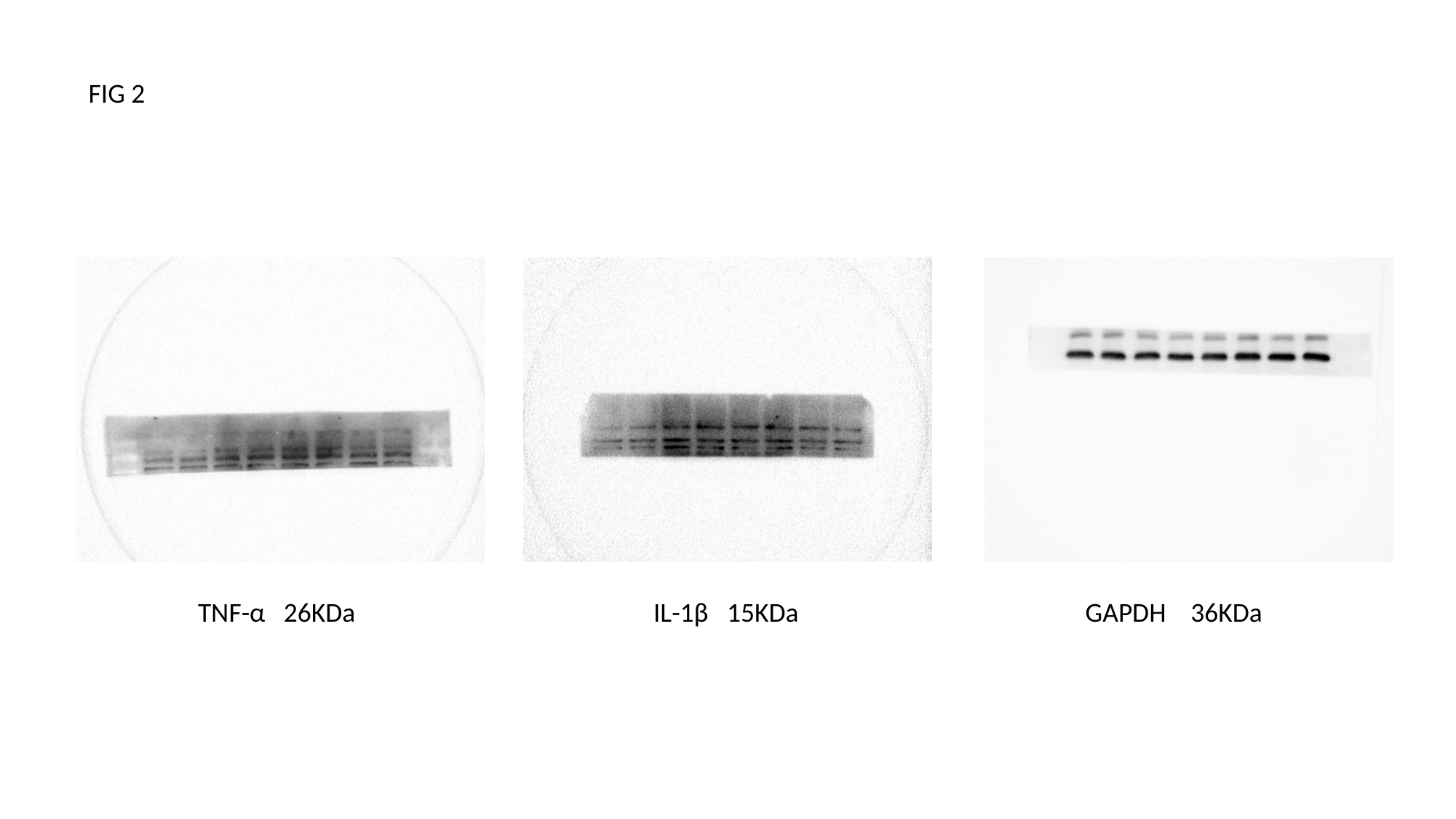

FIG 2
TNF-α 26KDa
IL-1β 15KDa
GAPDH 36KDa

## Slide 2
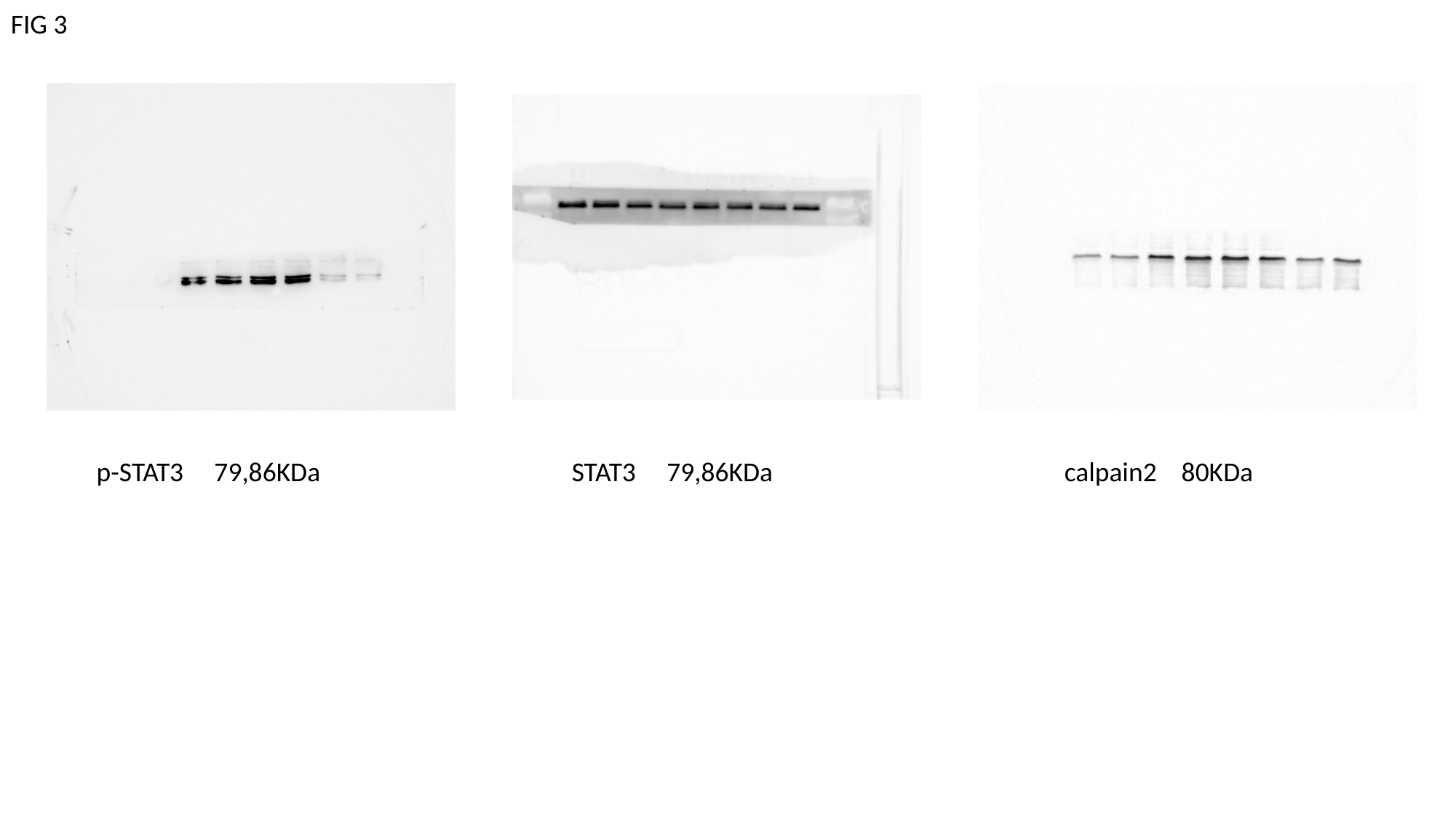

FIG 3
p-STAT3 79,86KDa
STAT3 79,86KDa
calpain2 80KDa

## Slide 3
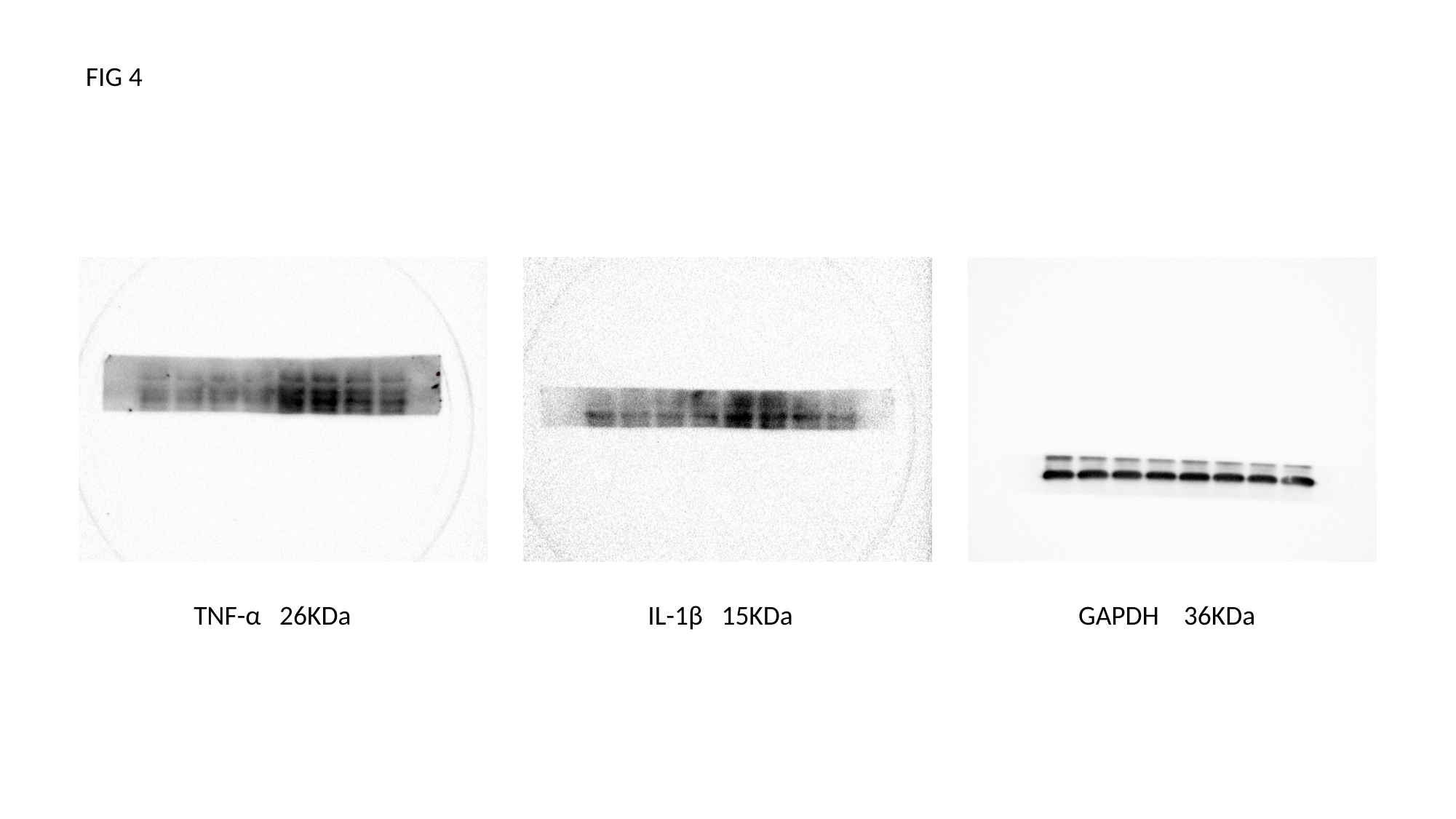

FIG 4
TNF-α 26KDa
IL-1β 15KDa
GAPDH 36KDa

## Slide 4
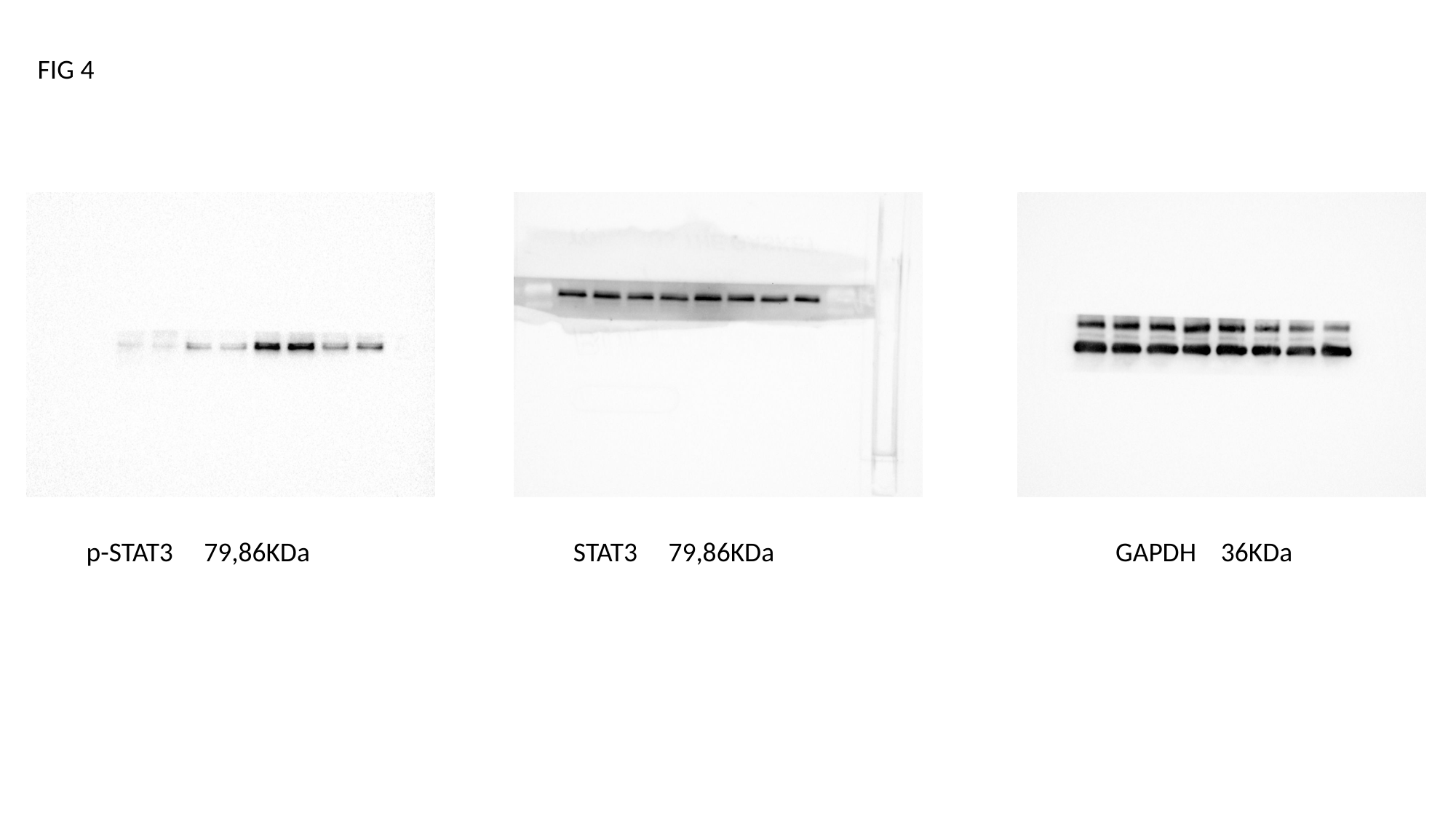

FIG 4
p-STAT3 79,86KDa
STAT3 79,86KDa
GAPDH 36KDa

## Slide 5
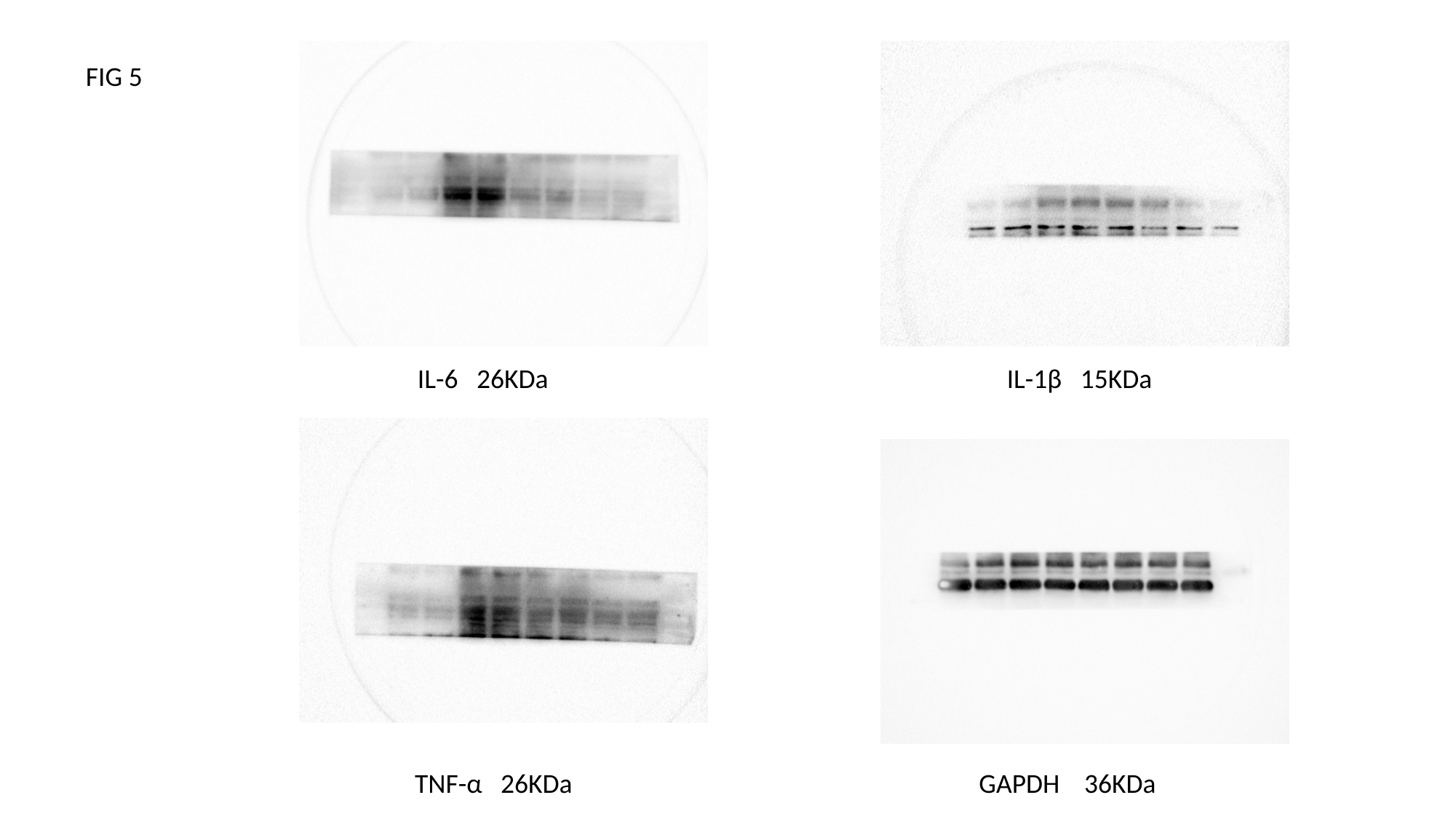

FIG 5
IL-6 26KDa
IL-1β 15KDa
TNF-α 26KDa
GAPDH 36KDa
